# Pre-Steady-State Decoding of the Bicoid Morphogen Gradient

**DOI:** 10.1371/journal.pbio.0050046

**Published:** 2007-02-06

**Authors:** Sven Bergmann, Oded Sandler, Hila Sberro, Sara Shnider, Eyal Schejter, Ben-Zion Shilo, Naama Barkai

**Affiliations:** 1 Department of Molecular Genetics, Weizmann Institute of Science, Rehovot, Israel; 2 Physics of Complex Systems, Weizmann Institute of Science, Rehovot, Israel; 3 Department of Medical Genetics, University of Lausanne, Lausanne, Switzerland; 4 Swiss Institute of Bioinformatics, Lausanne, Switzerland; Cambridge University, United Kingdom

## Abstract

Morphogen gradients are established by the localized production and subsequent diffusion of signaling molecules. It is generally assumed that cell fates are induced only after morphogen profiles have reached their steady state. Yet, patterning processes during early development occur rapidly, and tissue patterning may precede the convergence of the gradient to its steady state. Here we consider the implications of pre-steady-state decoding of the Bicoid morphogen gradient for patterning of the anterior–posterior axis of the *Drosophila* embryo. Quantitative analysis of the shift in the expression domains of several Bicoid targets (gap genes) upon alteration of *bcd* dosage, as well as a temporal analysis of a reporter for Bicoid activity, suggest that a transient decoding mechanism is employed in this setting. We show that decoding the pre-steady-state morphogen profile can reduce patterning errors caused by fluctuations in the rate of morphogen production. This can explain the surprisingly small shifts in gap and pair-rule gene expression domains observed in response to alterations in *bcd* dosage.

## Abstract 2

It was previously thought that cell fates were determined by morphogen gradients only after steady state was established. Here the authors show fate may precede gradient steady state.

## Author Summary

Subdivision of naive fields of cells into separate cell populations, distinguished by the unique combinations of genes they express, constitutes a major aspect of organism development. Classically, this involves the generation of gradients of signaling molecules (morphogens), which induce distinct cell fates in a concentration-dependent manner. It has been generally assumed that morphogen gradients are interpreted only after they reach a spatially fixed, steady-state profile. Our study re-examines this assumption for the classical case of the Bicoid morphogen, a transcription factor that is distributed as a gradient in the early *Drosophila* embryo. We propose and present evidence for dynamic, pre-steady-state decoding of the Bicoid profile. We further show that such dynamic decoding can directly account for the surprisingly small shifts in the expression domains of target genes, observed in response to altered Bicoid dosage, without invoking additional mechanisms or contributing factors. Pre-steady-state decoding can thus provide a simple explanation for the relative robustness of this classical morphogen system, which has been a long-standing problem.

## Introduction

Developmental patterning requires the translation of cell position into cell fate. In most prevalent models, positional information is provided by gradients of signaling molecules, called morphogens, which induce several cell fates in a concentration-dependent manner [1]. Prominent examples of such morphogens include members of the BMP, Wnt and Hh families of signaling molecules, which play a crucial role in patterning a broad spectrum of tissues and organisms [2–8]. While a variety of molecular mechanisms involved in the establishment of morphogen gradients have been described, the means by which these gradients are decoded are not well understood. In particular, little is known about the time at which the morphogen signal is being interpreted by its downstream targets. Most studies assume that the eventual pattern is defined according to the steady-state morphogen profile. Relying on the steady-state profile provides two obvious advantages. First, it allows for a temporal integration of a stable gradient, and as such may increase the readout fidelity. Second, it is relatively insensitive to the precise readout time and may thus compensate for perturbations that alter developmental timing.

Recent theoretical studies in several systems, however, predicted that the underlying cells respond to the pre-steady-state morphogen profile. For example, numerical simulations of Shh morphogen formation in the neural tube suggested that as soon as the morphogen signal increases above some threshold value, it can induce a given cell fate, implying that tissue patterning occurs before the morphogen concentration has reached its steady state [9]. Similarly, based on numerical simulations of mutant data, other authors argued in favor of pre-steady-state readouts in the gap gene interaction network [10] and of the BMP gradient [11] during early patterning of the *Drosophila* embryo. However, qualitative differences between pre-steady-state versus steady-state patterning, and their biological implications, have not been addressed.

A key aspect in developmental patterning is robustness: patterning is remarkably insensitive to fluctuations in the external environment or the precise genetic makeup. In fact, most genetic polymorphisms, or heterozygous mutations in developmentally related genes, have no detectable effect on patterning. Recent studies characterized feedback mechanisms that can be employed for shaping morphogen gradients and buffering their profile against fluctuations in gene dosage or environmental perturbations [12–21]. Most of the feedback mechanisms described require some time delay, and are most effective in steady state. In contrast, the robustness of decoding the pre-steady-state profile has not yet been examined.

The early patterning of the *Drosophila* embryo along its anterior–posterior axis serves as a classic example of morphogen-based patterning (see [22] for recent review). A principle morphogen in this system is Bicoid (Bcd), a transcription factor that is translated from maternally provided mRNA localized to the anterior pole of the embryo. The graded distribution of Bcd was visualized directly, providing the first molecular demonstration of a gradient of patterning molecules [23,24]. Bcd binds to the promoters of zygotic downstream genes (gap genes), and induces their expression in a concentration-dependent manner [25–30]. This Bcd-dependent induction, together with cross-interactions between the gap genes themselves, governs the patterning of the early embryo into distinct domains of gene expression [30]. Importantly, this patterning proceeds rapidly, with gap gene expression observed in less than 90 min after the onset of embryonic development [31].

Consistent with its presumed function as a morphogen, changes in *bcd* gene dosage shift the expression domains of gap genes such as *hunchback (hb)* [28,32] as well as the embryonic fate map [23]. Quantitative measurements, however, revealed that these shifts are significantly smaller than expected from a simple morphogen model [23,32]. For example, in embryos derived from mothers bearing only one functional allele of *bcd*, the Hb expression domain shifts by only ∼7% in embryo length (EL), about half of what is expected theoretically (see Equation 4 and [32,33]). Based on these apparent inconsistencies, it has been argued that the Bcd gradient is not sufficient for defining gap gene expression domains, and that an additional, and yet unknown, molecular mechanism is required to complement the Bcd gradient [32,33–36].

Here we report an analysis of the dynamics of gap gene determination. We found that the apparent anomalous shifts in gap gene expression domains can be readily explained if the gap genes start being expressed before the Bcd profile has reached its steady state. The interactions between gap genes can stabilize the initial gap expression domains, while the Bcd profile continues to expand. We show analytically that decoding the pre-steady-state profile enhances the robustness to changes in morphogen production rate. Two predictions of the pre-steady-state decoding were examined experimentally. First, we find that the observed shifts in gap gene expression domains depend on their position along the anterior–posterior axis, with more posterior positions being less sensitive to Bcd dosage. This result can be readily explained by a dynamic readout, but is inconsistent with decoding the steady state of the Bcd profile (compare with [15]). Second, using a reporter gene driven by a promoter containing Bcd-binding sites, we provide evidence that the Bcd profile still spreads out posteriorly at the times relevant for gap gene induction. Taken together, our results suggest that gap gene expression domains are defined by a transient, pre-steady-state Bcd profile. This pre-steady-state decoding reduces the sensitivity of the resulting pattern to changes in *bcd* dosage.

## Results

### Pre-Steady-State Decoding Can Explain the Anomalous Shifts in Gap Gene Expression Domains

Previous attempts to explain the anomalous shifts in Hb expression domains invoked maternally expressed Hb (mat-Hb) [37], predicting a significant contribution of mat-Hb to embryonic patterning. Indeed, translational repression of mat-Hb by the Nanos protein in the posterior part of the embryo establishes an anterior–posterior gradient of Hb protein [38–40], and Hb protein was shown to synergize with Bcd in patterning the embryo [41–44]. Yet, this proposal was found to be inconsistent, since gap gene expression domains in embryos derived from mat-Hb–deficient females are indistinguishable from wild-type embryos [32,38–40], reflecting the dominance of Bcd-dependent zygotic Hb expression over the contribution of mat-Hb (compare with Protocol S1). Alternative explanations invoked the existence of a secondary, yet to be identified morphogen gradient that is linked to Bcd (e.g., through the use of a common degradation machinery [33–36]).

Recently, a quantitative model of the gene network controlling gap gene expression was reported [45,46]. This model successfully reproduced the gap gene expression domains under wild-type conditions, and showed that gap gene patterning is a dynamic process to which the Bcd gradient only contributes the initial cue. Nevertheless, our reanalysis of this model for altered *bcd* dosage failed to reproduce the observed shifts of the gap gene expression domains (see Protocol S1). Since most model parameters, such as diffusion constants, transcription rates, or degradation rates, are not firmly established, we asked whether under a different set of assumptions, the gap gene network could still explain the lower than expected sensitivity of the gap gene expression domains to an altered *bcd* dosage.

**Figure 1:**
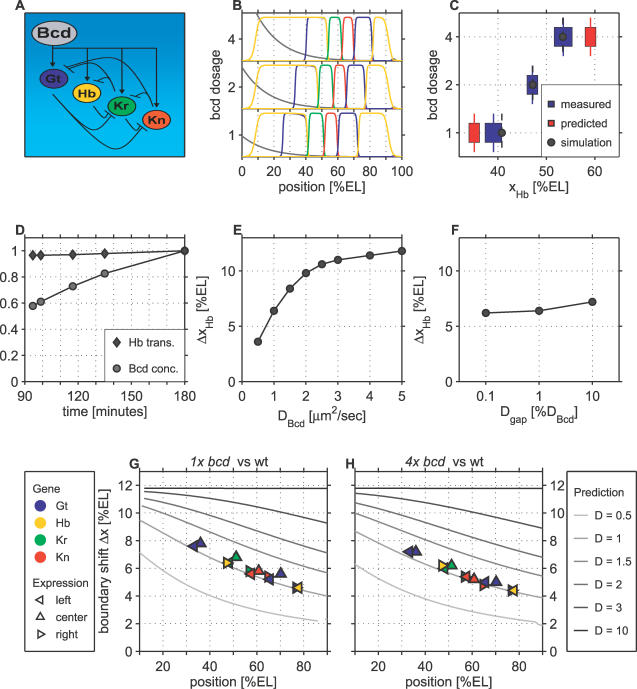
In Silico Simulation of the Gap Gene Network (A) A scheme of the gap gene interactions used: Bcd activates the expression of the gap genes in a concentration-dependent manner. Most gap genes mutually suppress each other (see Figure 9 in [45,46]). These interactions were modeled by a set of reaction diffusion equations, as specified in Protocol S1. (B) Spatial distributions of the different network proteins (color-coded as in [A]), at a time when the Bcd gradient has fully evolved and is close to exponential are shown for different *bcd* dosages. The gap genes are expressed in adjacent stripes, consistent with their in vivo expression domains. (C) The position of the Hb expression boundary in wild-type embryos (with 2 copies of *bcd*) and in embryos bearing altered *bcd* dosage (one and four copies, as shown). The results in our simulation (black circles) are compared to the experimental measurements (blue bars). Also shown is the prediction based on a steady-state gradient (red bars). (D) The temporal change in the position of the Hb expression boundary (diamonds) and in the Bcd concentration at this position (circles) are shown. We considered the wild-type situation (2 × *bcd*) and show the behavior following the initialization of gap gene expression. (E) We performed the simulations for different values of the Bcd diffusion constant *D*. Shown here is the shift of the Hb expression boundary in embryos with one *bcd* allele (1 × *bcd*) with respect to the wild-type as a function of *D*. (F) Same as in (E) but for different values of the gap gene diffusion constant. (G) Shifts of gap gene expression domains (center, left and right boundary, if applicable) in embryos with one functional *bcd* allele (1 × *bcd*) as a function of the wild-type (2 × *bcd*) position. (H) Same as in (G) but simulating embryos with four copies of *bcd* (4 × *bcd*).

Previous dynamic models considered a steady-state Bcd profile [33–37,45,46]. A recent review, however, emphasized the importance of assessing the dynamics of Bcd explicitly [47]. We have thus included these dynamics in our simulations. We considered the known interactions between Bcd and the gap genes, as well as the cross-interactions between the gap genes [30,45,46] (see Figure 1A and Protocol S1 for details of our in silico model). We assumed that translation of *bcd* mRNA is localized at the anterior pole of the embryo, and is initiated upon egg laying (defined as time *t* = 0). Zygotic gap gene expression begins at a later time (t_gap_ > 0), taken here as 90 min (corresponding to cycle 10 of the synchronous nuclear divisions of the early embryo, at 25 °C [31]. To characterize the model we first calculated the pattern of gap gene expression in wild-type embryos (with two *bcd* alleles), and compared these predictions with the experimentally determined patterns. We then measured the shift in the Hb expression domain upon a two-fold change in *bcd* gene dosage (corresponding to embryos whose mothers had either one or four functional *bcd* alleles).

The behavior of the model was analyzed for a range of realistic Bcd diffusion coefficients, as well as for different values of the parameters describing the gap gene network. A parameter regime was readily identified which reproduced both the wild-type pattern, as well as the experimentally observed shifts in Hb expression upon changes in *bcd* dosage (Figure 1B and 1C). Surprisingly, analysis of the relevant parameters suggested that reduced sensitivity to alterations in *bcd* gene dosage is achieved when pre-steady-state decoding is used: the Bcd profile at the onset of zygotic expression was still far from steady state (Figure 1D). Indeed, increasing the Bcd diffusion constant significantly enhances the sensitivity of Hb expression domains to *bcd* dosage (Figure 1E). Notably, the Hb expression domain, as well as those of the other gap genes, displayed only a small drift following their initial determination, although the Bcd profile continued to evolve (Figure 1D). This temporal stabilization of gap gene expression pattern is due to the mutual repression between adjacent gap genes and their limited diffusibility. Proper patterning can be achieved for a wide range of parameters, and the exact choice has only a marginal effect on the level of robustness (Figure 1F).

Our results thus indicate that the known interactions between the gap genes are sufficient to account for the phenotypes of embryos derived from mothers with altered *bcd* dosage. Previous studies, which concluded that the experimentally observed shifts in the Hb expression domain are inconsistent with a simple threshold model, calculated the expected shifts based on the assumption that the Bcd profile has reached a steady state [23,24,32]. In contrast, a significantly smaller shift is expected if decoding is executed before steady state has been reached.

### Enhanced Robustness of the Pre-Steady-State Profile: Mathematical Analysis

**Figure 2:**
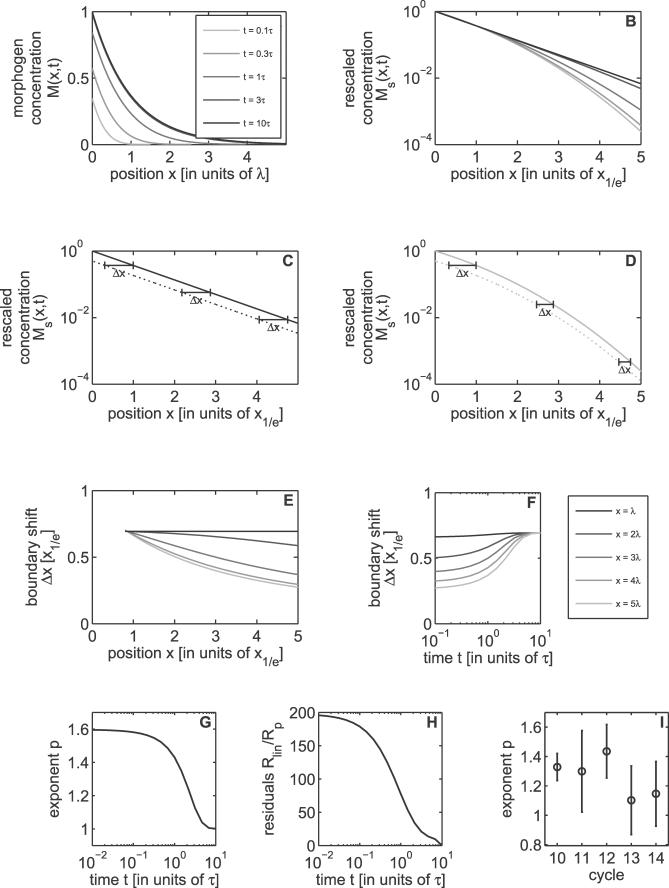
Properties of the Pre-Steady-State Morphogen Distribution (A) The morphogen distribution *M*(*x*, *t*) is plotted as a function of position *x* for different times *t* (legend). The plots were obtained by solving the reaction diffusion Equation 1 in one dimension (see Protocol S1). The position *x* is in units of the decay length scale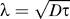 , while the time *t* is in units of the decay time *τ.* (B) Same as in (A), except that each profile was rescaled such that it has unit concentration at *x* = 0 and decays to 1/*e* at *x =* 1. Note the logarithmic scale. At early times, the profile tail decays super-exponentially, while at later times the morphogen distribution is well approximated by an exponential. (C) Alteration of the steady-state morphogen concentration upon 2-fold reduction in morphogen production rate. The original profile corresponds to the solid line and the altered profile to the dotted line. Note that the indicated positional shifts Δ*x = |x − x′|* at different morphogen thresholds do not depend on the position *x*. (D) Same as in (C) but for the pre-steady-state profile. (E) The shift Δ*x* is shown as a function of *x* for different times *t* (see legend in [A]). For pre-steady-state profiles, Δ*x* decreases as a function of *x*. (F) The shift Δ*x* as a function of time *t* is shown for different positions *x*, as indicated. While at late times the shift is almost independent of the position, at early times the shift decreases with increasing distance from the source. (G) The exact solution for the pre-steady-state profile was fitted to the phenomenological approximation *M_p_*(*x, t*) = *M*_0_(*t*) exp[−(*x*/*λ*(*t*))*^p^* ^(*t*)^]*.* The best-fitted exponent *p* is shown as a function of time (in units of the decay time *τ*). (H) To estimate the deviation of the time-dependent solution from an exponential, we compared the residual error obtained for the best-fit *p* approximation *(R_p_)* to the residual error obtained when fitting to exponential with *p =*1 (*R*_lin_). The ratio of these residual errors is shown as a function of time. (I) The best-fitted exponent *p* for quantitative Bcd profiles corresponding to wild-type embryos between cycles 10 and 14. Data were downloaded from the FlyEx database [56]. For embryos in cycles 10–12, the average *p* is significantly larger than 1, indicating superexponential decay, while Bcd profiles at cycles 13 and 14 are consistent with *p* = 1. Note, however, the large fluctuations.

Our numerical simulations indicate that the sensitivity to changes in Bcd dosage is lower when decoding is performed early, before steady state is reached. To better understand this result, we studied analytically the properties of the time-dependent morphogen profile. (Readers less interested in the mathematical details are encouraged to move directly to the next section.) We considered the canonical model of a morphogen system, applicable in the absence of feedback mechanisms affecting morphogen diffusion or degradation. The model postulates a single morphogen that diffuses in a naive field, where it is subject to uniform degradation. The time-dependent morphogen profile *M* (*x,t*) is obtained by solving the reaction-diffusion equation

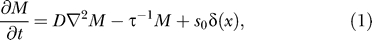
where *D* and *τ* denote the morphogen diffusion coefficient and degradation time, respectively. We assume that morphogen is produced at *x* = 0 at a constant rate s_0_. Equation 1 can be solved analytically (see Protocol S1 for derivation), giving

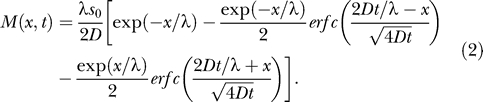
As shown in Figure 2A, morphogen spreads away from its source and assumes a more graded spatial distribution with time. At steady state, morphogen is distributed exponentially, *M*(*x*) = *M*_0_ exp(*−x/λ*), decaying over a typical length-scale 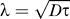 . Note that the time to reach steady state is controlled by the typical degradation time *τ*. At early times, *t *≪ *τ*, the system is still far from steady state, whereas for *t* ≫ *τ,* the morphogen gradient is close to steady state. Moreover, closer to the source the morphogen gradient approaches its steady state faster (Figure 2B).

To examine the robustness of the profile, we considered gene expression boundaries defined according to particular threshold levels of morphogen concentration. We then determined the position at which the morphogen level equals to that threshold. The exact shift in boundary position caused by a change in the morphogen production rate can be calculated numerically using Equation 2 (Figure 2C–2F; see also Protocol S1). To obtain analytical insight, however, it is instructive to consider the following phenomenological approximation for the time-dependent morphogen profile

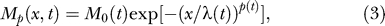
where *M*_0_(*t*) is proportional to the morphogen production rate. In this approximation, the exponent *p*(*t*) decreases monotonically with time. For a pulse-like morphogen production, the morphogen distribution at short times (*t* ≪ *τ*) resembles a Gaussian distribution, corresponding to *p*(*t*) = 2 and 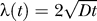 (see Protocol S1). When production is continuous, the short-time distribution is better approximated by a smaller exponent, *p*(*t*) ≈ 1.6 (Figure 2G). At longer times (*t* ≫ *τ*), the distribution approaches an exponential profile, corresponding to *p*(*t*) = 1 and *λ*(*t*) *= λ*.

Within this approximation, the computation of the shift in the boundary position is straightforward. Suppose that the morphogen-production rate is altered by a factor *γ*. We can approximate the position-dependent shift in morphogen profile as (see Protocol S1)

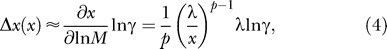
with *p* = *p*(*t*) and *λ* = *λ*(*t*)*.* Clearly, for *x ≥ λ,* the magnitude of the shift Δ*x* increases with decreasing *p* > 1. Equation 4 demonstrates that in most of the field (*x > λ*)*,* the shift in boundary position increases with decreasing *p.* Since *p* decreases in time toward its minimal steady-state value (*p* = 1), a greater degree of robustness is achieved at earlier times, before steady state is reached. This result also holds for the exact solution in Equation 2 (see Figure 2F for numerical analysis). In fact, the system is most sensitive when cell fate boundaries are defined according to the steady-state morphogen profile. Thus, the capacity to buffer fluctuations in morphogen production rate is enhanced if decoding is executed early, when the gradient is still far from steady state.

Notably, at steady state (*p* = 1), the shift Δ*x* is predicted to be independent of the position *x*. Thus, a uniform shift independent of the distance is a hallmark for the decoding of a steady-state profile. Indeed, as we have shown previously [15], this result holds not only for the canonical model studied here, but for any decoding of a single morphogen steady-state gradient also in the presence of arbitrary feedback mechanisms affecting morphogen diffusion or degradation. In contrast, when decoding is based on the transient, pre-steady-state morphogen levels (*p* > 1), the magnitude of the induced shift is position dependent, and decreases with increasing distance from the source. Again, this effect is seen also in the numerical analysis of the full solution in Equation 2 (Figure 2D–2F).

### The Spatial Pattern of Gap Gene Sensitivity Is Consistent with Pre-Steady-State but Not Steady-State Decoding

To further examine the possibility that gap gene expression domains are defined by the pre-steady-state Bcd profile, we searched for properties that distinguish between steady-state and pre-steady-state decoding strategies. This search was guided by our mathematical analysis of the canonical morphogen model that takes into account the spatiotemporal formation of the morphogen gradient (see above). Briefly, we considered perturbations to the morphogen production rate, and analyzed the resulting shifts in expression patterns predicted by this model when decoding is performed at different timepoints following the initiation of morphogen production. The most prominent distinction between steady-state versus pre-steady-state decoding that we observed was that in the case of steady-state decoding, the extent of the shift in an expression domain was independent of the spatial position of this domain (Figure 2C). (This result is valid for the decoding of any steady-state profile of a single diffusing morphogen even in the presence of feedback, cooperativity, or other form of nonlinearity; compare with [15].) In contrast, when decoding was based on transient, pre-steady-state morphogen levels, the magnitude of the induced shift was position dependent, decreasing with increasing distance from the source (Figure 2D and 2E).

**Figure 3:**
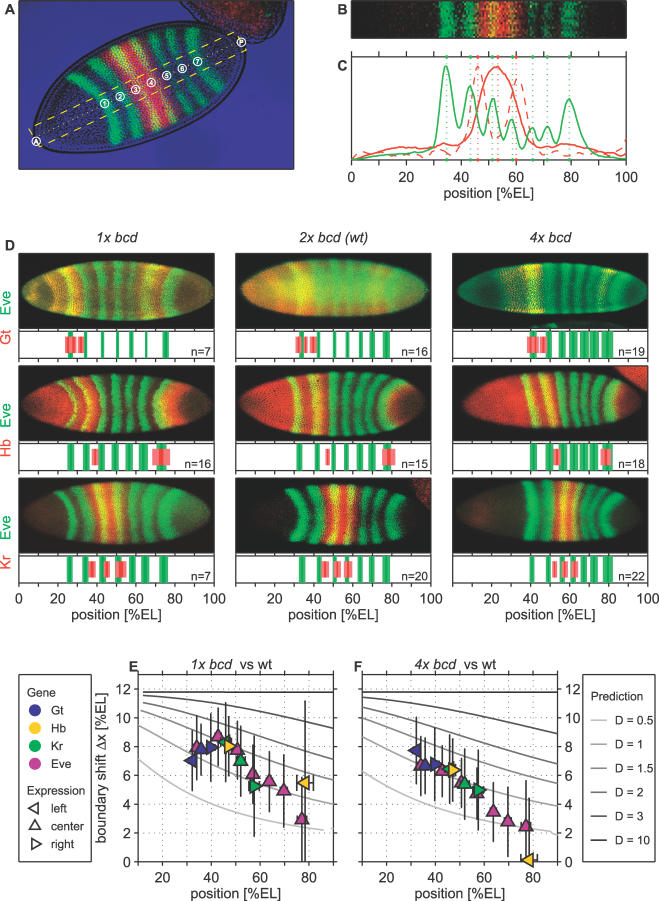
Quantitative Effects of Altered Maternal *bcd* Gene Dosage on Zygotic Target Gene Expression (A) Dorsal view of a representative cycle-14 wild-type *Drosophila* embryo stained for the Eve (green) and Kr (red) proteins. The contours of the embryo were determined from the transmitted light image (blue). (B) (C) Quantitative analysis of stripe positions was performed by semiautomated software as follows: the positions of the embryo poles and of the first and last Eve stripes were defined manually. Based on these definitions, a rectangular area (yellow dashed in [A]) corresponding in height to 10% EL was extracted automatically. Intensity profiles (solid lines in [C]) were obtained by averaging the fluorescence signal along the dorsal–ventral axis in this area and subsequent smoothing. Stripe positions (green dotted lines in [C]) and boundaries (red dotted lines in [C]) were defined based on local maxima of these profiles and their first derivative, respectively. (D) Expression domains of Eve (green) and the gap genes *Gt, Hb,* or *Kr* (red; as indicated) in embryos derived from females bearing one, two, or four copies of *bcd.* In each panel, the top part displays a representative confocal image, while quantitative results obtained from multiple embryos are shown at the bottom part. The widths of the stripes correspond to the standard errors (bright) and deviations (shaded). *n* denotes the number of embryos used in each analysis. (E) Observed shifts of target gene expression domains (center, left, and right boundaries, if applicable) in embryos with one functional *bcd* allele (1 × *bcd*) as a function of the wild-type (2 × *bcd*) position. Gray lines indicate theoretical predictions for different Bcd decay times (compare with Figure 1G and 1H). (F) Same as in (G) but for embryos with four copies of *bcd* (4 × *bcd*).

To examine which of these two behaviors is observed in early *Drosophila* embryos, we measured the shifts of different gap gene expression domains induced by altered *bcd* gene dosage. We examined embryos derived from mothers carrying only one functional *bcd* allele, as well as embryos derived from wild-type females (bearing two functional alleles) and from females that carry two additional (total of four) functional *bcd* alleles. Using existing antibodies [48], we stained these embryos for the protein products of several gap genes *(hb, Kruppel [Kr],* or *giant [gt]),* and for the downstream pair-rule gene even-skipped *(eve),* whose expression is gap gene dependent. Automated image processing was used to determine expression domain boundaries (Figure 3A–3D). As reported previously, when the maternal *bcd* dosage was reduced to one copy, all *bcd*-dependent expression domains were shifted towards the anterior part of the embryo, while increasing maternal *bcd* dosage to four copies resulted in shifting of all domains towards the posterior end [23,32].

Yet, the extent of the shifts in gap gene expression domains was not uniform, but decreased towards the posterior pole, such that expression domains closer to the source were more strongly affected by changes in *bcd* dosage (Figure 3E and 3F). Moreover, the measured shifts in midembryo positions were generally consistent with those obtained in numerical simulations based on pre-steady-state decoding and a Bcd diffusion constant *D* ∼ 1 μm^2^/s (gray lines in Figure 3E and 3F; compare also Figure 1G and 1H). Deviations from the predicted shifts were observed for posterior expression domains (e.g., posterior Hb), probably reflecting their dependence on the terminal gap genes *tailless* and *huckebein* [49–51], and on maternally provided *caudal* [30,52,53].

### A Reporter Driven by Bcd-Binding Sites Does Not Reach a Steady State at the Beginning of Gap Gene Expression

As a more direct test of the pre-steady-state decoding strategy, we wanted to follow temporal changes in the Bcd profile itself, at a time when gap gene expression domains are first defined. Gap gene expression is clearly observed at division cycle 10 (∼90 min after egg lay at 25 °C), with some reports suggesting that it is initiated as early as cycle 8 (∼20 min earlier; see [31] and references therein). Recent analyses have identified a similar time window (65–100 min after fertilization) as the critical time for perturbing gap gene expression domains [54]. Moreover, degradation of *bcd* mRNA is initiated at cycle 12 [55], further pointing to cycles 10–11 as relevant for gap gene determination. Examining anti-Bcd staining images from the FlyEx database [56] we observed that Bcd profiles in cycles 10–12 appear to have not yet reached an exponential shape (see Figure 2I).

Direct immunological quantification of the Bcd profile at early stages is difficult, however, since existing antibodies provide low and variable staining intensity. To overcome this limitation, we resorted to a functional assay using a Bcd-responsive reporter. For this assay we chose to use hb123x3-lacZ, in which lacZ reporter expression is under the control of a triplicated fragment of 123 bp derived from the *hb* promoter, containing multiple, functional Bcd-binding sites [28]. An added benefit of this approach is the sharp transition of reporter expression, which facilitates the determination of the transcription domain regardless of the absolute level of expression. Transgenic flies bearing multiple copies of this reporter were generated as a means to increase the signal. The expression pattern of this reporter was previously shown to faithfully reflect Bcd activity, and to bind Bcd directly [28]. Although we cannot completely rule out binding of additional factors to this short element, we note that this reporter also maintains its precise expression domain in the absence of zygotic Hb [28]. Since the removal of zygotic Hb was shown to shift the adjacent Kruppel and Knirps expression domains [41], it is unlikely that the expression domain of the reporter element we are using is influenced by cardinal gap genes.

**Figure 4:**
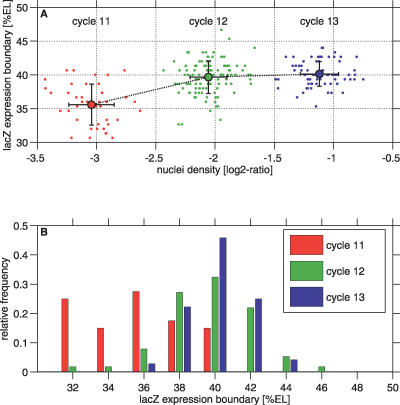
Analysis of Bcd-Dependent *lac*Z Reporter Expression over Cleavage Cycles 11, 12, and 13 The posterior boundary of the *lac*Z expression domain is shown as a function of the normalized nuclear density for each embryo (colored dots). Embryos fall into three classes of nuclear density corresponding to their cleavage cycle (11, red; 12, green; and 13, blue). Average nuclear density and domain boundary for each cycle are indicated by big circles, and whiskers denote standard deviations. (B) The distribution of the expression boundary is shown for the three cycles (bin size is 2% EL). Note the progression in time of the boundary.

In situ hybridization to mRNA provides a sensitive readout of reporter expression. Expression of lacZ mRNA could be observed in embryos bearing the reporter beginning at cycle 11. The total level of expression increased with time due to the elevated efficiency of zygotic gene expression in subsequent cycles and the increase in the number of nuclei. A significant shift in the posterior boundary of the lacZ mRNA expression domain was observed between cycles 11 and 12 (Figure 4), with the lacZ expression domain in cycle 12 embryos positioned ∼10% more posteriorly, on average, compared to cycle 11 embryos. Posterior progression was observed only until cycle 13, probably due to degradation of bcd mRNA. However, the clear shift between cycles 11 and 12 is consistent with the proposal that the Bcd profile is still far from its steady state at the relevant timeframe for decoding.

## Discussion

Subdivision of the early *Drosophila* embryo into distinct domains of gap gene expression is arguably the best-studied paradigm of morphogen-induced patterning. Despite extensive investigation, however, quantitative properties of this system have proven difficult to explain, prompting the proposition that additional yet unknown molecules or mechanisms are yet to be identified. The lower-than-expected sensitivity of the pattern to *bcd* gene dosage is one such mystery, noted repeatedly in studies characterizing the Bcd gradient [23,32,37]. Our study shows, however, that this property can be readily explained within the known framework of the gap gene expression network, by assuming that gap genes begin to be expressed before the Bcd profile has reached its steady state.

More generally, we have shown that pre-steady-state decoding of morphogen gradients can enhance robustness to changes in the rate of morphogen production. This result was derived within the canonical model of morphogen gradient formation, assuming that no feedback mechanisms exist that alter the diffusion or degradation of the morphogen molecules. In previous attempts to explain robustness of patterning, we and others have focused on the steady-state distribution, describing feedback mechanisms that reduce gene dosage sensitivity [12–21]. For example, we have shown that self-enhanced degradation can ensure high robustness with respect to fluctuations in morphogen production rate [15]. However, such feedback mechanisms typically rely on lengthy transcription or translation, and their applicability to early development, where patterning is rapid, is questionable. Pre-steady-state decoding may provide a compelling alternative for increasing robustness, without the need for any explicit feedbacks.

Pre-steady-state decoding assumes that cell fates are defined before the morphogen profile has reached its steady state. The time to reach steady state is controlled by the morphogen decay time *τ*. In particular, enhanced robustness will be apparent for times that are lower, or comparable to *τ*. For example, the experimentally measured shifts of gap gene expression domains are consistent with a decoding time *t*_gap_ that is equal to this decay time, *t*_gap_ = *τ*. Interestingly, since the rate of convergence to steady state at a particular position *x* increases with the distance to the source, anterior regions will appear rather close to their steady state even at *t* = *τ*. For example, within our simple model (Equation 2) for *t* = *τ* the profile at *x* = *λ* /2 (∼50 μm) has already exceeded 75% of its steady-state value, whereas at *x* = *2λ* (∼200 mm) it is still below 40% of its final value (see Figure S1).

Thus, pre-steady-state decoding will be valid if the Bcd degradation time is not shorter than ∼60–90 min, the time when gap gene expression is first observed [31]. Although the Bcd degradation time was not yet measured, a lower bound for this time can be estimated using the measured Bcd profile at cycle 14, which has a decay length of λ ∼ 100 μm. A steady-state profile that extends to this range requires a decay time τ** =** *λ^2^*/*D* ∼ 170/*D* minutes, where *D* is the Bcd diffusion constant (in units of μm^2^/s). It was recently reported that biologically inert Dextran molecules with a molecular mass comparable to that of Bcd, diffuse quite rapidly in the early *Drosophila* embryo, with *D* ∼ 17 μm^2^/s [57]. This value, if applicable also to Bcd, would imply a short decay time of ∼10 min, such that a steady-state profile is reached before decoding. However, biologically active molecules are known to diffuse at significantly slower rates within cellular environments, with measurements consistently finding diffusion constants in the range of 0.3–3 μm^2^/s [58–63]. Such values are not consistent with steady-state decoding. They provide a lower bound of 1–10 h for the Bcd decay time, and strongly support the notion of pre-steady-state decoding.

A key issue in pre-steady-state decoding is the definition and execution of a distinct time of decoding. Crucial questions at the mechanistic level are how the decoding time is defined and whether decoding is executed simultaneously in all parts of the embryo. One possibility is that the profile simply does not reach a steady state during the relevant developmental window of morphogen production and signaling. Since development is an ongoing dynamic process, the response to any given morphogen signal is limited to a specific time window, independently of whether the profile has reached its steady state or not. Accordingly, cell fate determination often involves an irreversible transition (commitment), rendering gene expression independent of the inducing signal. A second, conceptually related possibility is that gene expression is determined during the expansion of the morphogen profile, and loses its sensitivity to further changes in this profile. A recent model of neural tube patterning in vertebrates described such a mechanism, showing that a sharp boundary of gene expression is elicited early on in the evolution of the Shh gradient. Self-reinforcing interactions maintain the boundary spatial position even as the Shh gradient itself evolves, moving past the location where the boundary was initially specified [9].

In the case of the gap gene expression domains, the repression between adjacent gap genes can function to stabilize the pattern once it is formed. The gap genes first begin to be expressed at cycles 9–10, when all nuclei reach the periphery, capturing the early Bcd profile. Once gap genes expression is initiated, however, mutual repression between adjacent gap genes stabilizes their spatial expression pattern, and it remains fixed despite further evolution of the Bcd profile. Consistent with this scenario, Yucel and Small have recently argued that measurements of the Bcd gradient during late blastoderm stage (cycle 14) may not accurately reflect the shape of the gradient that defines the gap gene expression domains [47]. Indeed, *bcd* mRNA starts to degrade at cycle 12 [55], and its protein levels begin to fade [24,55]. Moreover, at cycle 14, gene expression becomes a combination of Bcd-dependent and Bcd-independent activation [47].

The increasing number of nuclei could also function to slow down Bcd diffusion and maintain its pre-steady-state profile. Bcd synthesis is initiated at egg lay, when the embryo consists of a single cell with a single nuclei, and continues throughout the initial set of 14 syncytial nuclear divisions, which increase the number of nuclei to ∼6,000. Interaction of Bcd with the nuclei, or with the cytoplasm surrounding the nuclei, can function to slow down its kinetics, effectively scaling the time to reach steady-state with the number of nuclei (see Protocol S1). Importantly, this apparent stabilization changes the effective time scale of the profile evolution, but does not alter its pre-steady-state characteristics.

By showing that the relative insensitivity of the gap gene expression domains to *bcd* dosage can be readily accounted for by pre-steady-state decoding, our study provides a simple and parsimonious explanation of one of the long-standing mysteries of the Bcd morphogen system. Additional quantitative issues such as the robust scaling with EL [32,64] and the apparent insensitivity to temperature [54] still remain unexplained. These unresolved issues are similarly likely to reveal new features of the Bcd patterning system.

## Materials and Methods

### *Drosophila* genetics.

Females bearing the normal two copies of the *bcd* gene were used as the wild-type strain. The *bcd* gene dosage was doubled (4 × *bcd*) in female progeny of a cross between yw females and P(bcd+5+8) males, a strain which harbors two additional, transgenic copies of the *bcd* gene on the X chromosome [19]. Females heterozygous for a chromosomal deficiency encompassing the *bcd* locus, and thus bearing only a single copy of the *bcd* gene (1 × *bcd*), were derived from a cross between Df(3R)MAP117/TM3 and *yw.*

### Embryo immunostaining.

Eggs 2–4 h old laid by females bearing various dosages of *bcd* and crossed to *yw* males were collected on agar plates at 25 °C. Egg fixation (3.5% formaldehyde) and preparation for immunostaining were according to standard protocols [65]. Immunostaining of fixed embryos was carried out for 16 h at 4 °C using the following primary antibodies: guinea pig anti-Hb (diluted 1:300), guinea pig anti-Kr (1:300), rabbit anti-Gt (1:500), and rabbit anti-Eve (1:500). Secondary antibodies (Cy3-anti-guinea pig, Cy2-anti-rabbit, and Cy3-anti-rabbit; Jackson Laboratories, http://www.jax.org) were applied for 2 h at room temperature. Double stainings were performed simultaneously, except for Gt/Eve, which was performed sequentially, since both primary antibodies are derived from rabbits.

### Microscopy and image analysis.

Images were obtained using a Bio-Rad Laboratories MRC-1024 confocal system (http://www.bio-rad.com), utilizing an argon-krypton mixed-gas laser and mounted on a Zeiss Axiovert microscope (http://www.zeiss.com). Analysis was restricted to cellularizing embryos displaying a distinct seven-stripe Eve pattern. Images were imported and analyzed using software programmed in Matlab (MathWorks, http://www.mathworks.com).

### Monitoring the Bcd activity gradient over time.

A fragment containing three copies of a 123 bp Bcd-responsive element from the *hb* promoter (hb123x3-lacZ; [28]) was inserted into the pH-Pelican lacZ reporter [66] and used to generate transgenic lines. To maximize the signal, a fly line homozygous for a chromosome carrying two insertions of the hb123x3-lacZ construct was used. To detect expression of the reporter construct, RNA in situ hybridization was performed with a lacZ RNA probe according to the protocol specified in http://superfly.ucsd.edu/∼davek/intro.html, with hybridization temperature of 65 °C. In addition, a 1:50 dilution of NBT/BCIP (catalog number 1,681,451; Roche, http://www.roche.com) was used as a substrate for alkaline phosphatase. To detect the nuclei, embryos were then incubated for 30 min with a 1:200 dilution of the nuclear dye TOPRO (Molecular Probes, http://probes.invitrogen.com) following treatment with RNAse (10 μg/ml for 30 min). To dynamically monitor the Bcd gradient, the location of the transition point from the signal zone to nonsignal zone of the lacZ gradient was measured in embryos of different ages with different nuclear densities. Microscopic images were obtained using a Bio-Rad Radiance 2100 confocal system. A transmitted light image of the lacZ gradient was obtained in the midfocal plane of a given embryo, and nuclei were viewed by fluorescence of the TOPRO dye. The two corresponding sets of images were analyzed with automated image processing tools developed in MatLab in order to measure the lacZ transition point and the nuclear density as proxy for the embryo's age (Protocol S1). Staining was first detected in embryos at cycle 11, and its intensity increased with embryo stage. We were worried that the increase in staining intensity could lead to the apparent shift in the transition point. This could be the case, for example, if the reporter expression boundaries remained in fact unchanged between different stages, but the minimal expression level cannot be detected at early stages due to low staining intensity. In this case the transition point (determined at half value between minimal and maximal intensity) would actually move posteriorly with increasing total intensity. To control for this possibility, we measured also the spatial distance over which the profile decayed from 80% to 20%. If the changes in transition points are due to changes in staining intensity, the distance over which the profile decays is also expected to increase with staining intensity. In contrast, we observed that the distance over which the profile decays in fact decreases with increasing staining intensity. Thus, it is unlikely that the shift in transition point is due to lower staining intensity at earlier times.

## Supporting Information

Protocol S1 Supplementary Information (1.1 MB PDF)

## Abbreviations

Bcd: Bicoid
EL: embryo length
mat-Hb: maternally expressed Hb
