# Supplementary material for: Pre-Steady-State Decoding of the Bicoid Morphogen Gradient
Source: PLoS Biol. 2007 Feb 6;5(2):e46. doi: 10.1371/journal.pbio.0050046 (PMC1790957; doi:10.1371/journal.pbio.0050046)
Supplement: Supplementary file 1 [file pbio.0050046.sd001.pdf]

Supplementary Information

Pre-steady state decoding of the Bicoid morphogen gradient

Sven Bergmann[#*&], Oded Sandler[#], Hila Sberro[#], Sara Shnider[#&], Ben-Zion Shilo[#], Eyal Schejter[#] and Naama Barkai[#&]

[#]Departments of Molecular Genetics and [&]Physics of Complex Systems
Weizmann Institute of Science, Rehovot 76100, Israel
[*]Department of Medical Genetics, University of Lausanne,
and Swiss Institute of Bioinformatics, Switzerland

November 27, 2006

Abstract

This text discusses in detail the time dependent morphogen profile in the presence of diffusion and degradation. We present the analytical solution for a one dimensional system with linear degradation. The limits of this solution for no degradation and for the steady state are discussed. We analyze the shift of a threshold position induced by a change in the morphogen production rate, both for the analytical solution in 1d and a numerical solution for a two-dimensional field of elliptical shape. We describe our re-analysis of the gene-circuit models and present our own dynamic simulation of gap-gene network. We also discuss the effect of morphogen binding to nuclear receptors in the context our model. Finally we provide some technical information on the image processing tools we developed for embryo image analysis.

Contents

1 Reaction-diffusion equation and solution **2**

2 Limiting cases **4**
2.1 No degradation 4
2.2 Steady state solution 5

3 Robustness of threshold position **5**

4 Numerical solutions **6**
4.1 The time-dependent morphogen gradient in two dimensions 6
4.2 Previous Simulations of dynamic evolution of gap gene expression 7
4.3 Our simulations for gap gene expression in response to a time-dependent Bcd gradient 8

5 The effect of morphogen binding **10**

6 Details on transition point and nuclei density measurements **11**

1 Reaction-diffusion equation and solution

In the following we derive explicitly the time-dependent solution for a single-morphogen system with linear degradation in one dimension. Such a system is described by the reaction-diffusion equation

$$\frac{\partial}{\partial t}M(x,t) = D\frac{\partial^2}{\partial x^2}M(x,t) - \alpha M(x,t) + s(x,t)\,, \tag{1}$$

where $M(x,t)$ denotes the morphogen concentration as a function of one spacial coordinate x and time t. $s(x,t)$ refers to a general source term. Note that eq. (1) contains two constants. The diffusion constant D has dimension $[D] = length^2/time$. The degradation rate α is the inverse of the decay time τ and has dimension $[\alpha] = 1/time$. Combining both constants we can define

$$\beta \equiv \sqrt{\alpha/D}\,, \tag{2}$$

which is the inverse of the decay length λ and has dimension $[\beta] = 1/length$.

Here we consider a source that is switched on at $t = 0$ and localized at the origin:

$$s(x,t) = s_0\delta(x)\Theta(t)\,. \tag{3}$$

Then, eq. (1) can be solved analytically, giving:

$$M(x,t) = \frac{s_0}{2\beta D}\, m(x,t) \tag{4}$$

where

$$m(x,t) \equiv \left[e^{-\beta x} - \frac{e^{-\beta x}}{2}\,\mathrm{erfc}\left(\frac{2\beta Dt - x}{\sqrt{4Dt}}\right) - \frac{e^{\beta x}}{2}\,\mathrm{erfc}\left(\frac{2\beta Dt + x}{\sqrt{4Dt}}\right)\right]. \tag{5}$$

Note that this solution only changes by a factor of two when diffusion into the region $x < 0$ is excluded, owing to the symmetry of the problem. One way to obtain the solution in eq. (4) is to consider the Fourier transformed morphogen density

$$\tilde{M}(k,\omega) \equiv \int_{-\infty}^{\infty} M(x,t)\, e^{-i(kx-\omega t)} dxdt\,. \tag{6}$$

In terms of $\tilde{M}(k,\omega)$ the reaction diffusion reaction (1)reads

$$i\omega\tilde{M}(k,\omega) = -(Dk^2+\alpha)\,\tilde{M}(k,\omega) + \tilde{s}(k,\omega)\,. \tag{7}$$

In our case the Fourier transform of the source term is

$$\tilde{s}(k,\omega) \equiv \int_{-\infty}^{\infty} s(x,t)\, e^{-i(kx-\omega t)} dxdt = s_0 \int_0^{\infty} e^{i\omega t} dt = \frac{is_0}{\omega}\,. \tag{8}$$

Solving for $\tilde{M}$ gives

$$\tilde{M}(k,\omega) = \frac{-s_0}{i\omega\,(i\omega + Dk^2 + \alpha)} = \frac{-s_0}{Dk^2+\alpha}\left(\frac{1}{i\omega} - \frac{1}{i\omega + Dk^2 + \alpha}\right). \tag{9}$$

The inverse Fourier transform of this yields $M(x,t)$. It is useful to compute first

$$\hat{M}(k,t) \equiv \frac{1}{2\pi}\int_{-\infty}^{\infty} \tilde{M}(k,\omega)\, e^{-i\omega t} d\omega = \frac{s_0}{Dk^2+\alpha}\left[1 - e^{-(Dk^2+\alpha)t}\right]\Theta(t)\,. \tag{10}$$

From $\hat{M}(k,t)$ the solution in eq. (4) is obtained by noting that the inverse Fourier transform of $1/(k^2+\alpha/D)$ is $\exp(-\sqrt{\alpha/D}|x|)/\sqrt{4\alpha/D}$ to compute the first term, and using the folding theorem,

$$\int_{-\infty}^{\infty} f_1(x) f_2(x)\, e^{-ikx} dx = \int_{-\infty}^{\infty} \tilde{f}_2(k')\tilde{f}_1(k-k') dk'\,, \tag{11}$$

where $\tilde{f}$ refer to the fourier transform of f, to compute the second term.

The time dependent formation of the morphogen gradient is shown in Fig. 2ab of the main paper. A crucial property of this formation is that close to the source (at $x = 0$) the gradient approaches its steady state value much earlier than far away from the source (Suppl. Fig. 1).

2 Limiting cases

Since the exact solution in eq. (4) has no simple form we introduce the phenomenological approximation

$$M_p(x) = M_0(t)\exp[-(x/\lambda(t))^{p(t)}] \tag{12}$$

in order to discuss the temporal behavior of the morphogen gradient (Eq. 2 in Box 1). In particular, the exponent $p(t)$ allows for an intuitive matching with the more familiar cases of negligible morphogen degradation (corresponding to very early times) and the steady state (corresponding to late times). We discuss both limits in the following.

2.1 No degradation

In the limit where $\alpha \to 0$ (and thus $\beta \to 0$ if D is constant) the system is determined by

$$\frac{d}{dt}M(x,t) = D\frac{\partial^2}{\partial x^2}M(x,t) + s(x,t)\,, \tag{13}$$

and has no fixed scale of length or time. Rather the typical length scale $\lambda(t) \equiv \sqrt{Dt}$ depends on the time (or the typical time scale $\tau(t) \equiv x^2/D$ depends on the position).

The solution to eq. (13), for $s(x,t)$ as in eq. (3),

$$M(x,t)|_{\alpha\to 0} = \frac{s_0}{2D}\left[\sqrt{\frac{4Dt}{\pi}}\,e^{-\frac{x^2}{4Dt}} - x\,\mathrm{erfc}\left(\frac{x}{\sqrt{4Dt}}\right)\right]\,, \tag{14}$$

can be obtained by solving eq. (13) first for $s(x,t) = s_0\delta(x)\delta(t)$, giving

$$M_0(x,t) = \frac{s_0}{\sqrt{4\pi Dt}}\,e^{-\frac{x^2}{4Dt}}\,, \tag{15}$$

and then integrating over t (i.e. $M(x,t)|_{\alpha\to 0} = \int_0^t M_0(x,t')dt'$). (Note that although the integrand has zero slope at $x = 0$ for all $t > 0$, this is not true for$M(x,t)|_{\alpha\to 0}$.) Alternatively, one can take the limit $\beta \to 0$ (which is equivalent to $\alpha \to 0$) of the full solution in eq. (4). To this end we expand

$$m(x,t) \simeq \underbrace{m(x,t)|_{\beta=0}}_{=0} + \left.\frac{\partial m(x,t)}{\partial\beta}\right|_{\beta=0}\cdot\beta = \beta\left[\sqrt{\frac{4Dt}{\pi}}\,e^{-\frac{x^2}{4Dt}} - x\,\mathrm{erfc}\left(\frac{x}{\sqrt{4Dt}}\right)\right]\,, \tag{16}$$

from which one obtains eq. (14) immediately.

To study the tail of this solution we note that

$$\mathrm{erfc}(z) \simeq \frac{1}{\sqrt{\pi}}\frac{e^{-z^2}}{z}\left(1 - \frac{1}{2z^2}\right) \quad \text{for } z \gg 1\,. \tag{17}$$

(This expansion can be obtained by repeatedly integrating $\mathrm{erfc}(z) = 2/\sqrt{\pi} \int_z^\infty \exp(-z'^2) dz'$ by parts.) Using eq. (17) we obtain

$$M(x,t)|_{\alpha\to 0} \simeq \frac{2s_0(Dt)^{3/2}}{\sqrt{\pi}D} \frac{e^{-\frac{x^2}{4Dt}}}{x^2} \quad \text{for } x \gg \lambda(t)\,. \tag{18}$$

Thus, asymptotically $\log(M(x,t)|_{\alpha\to 0}) \propto -x^2$ up to logarithmic corrections. Nevertheless, including the range where $x \sim \lambda(t)$, the best approximation to the solution in eq. (14) in terms of the approximation $M_p(x,t)$ in eq. (12) is achieved with $p \approx 1.6$ rather than $p = 2$.

2.2 Steady state solution

The steady state solution,

$$M_s(x) = \frac{s_0}{2\beta D} e^{-\beta x}\,, \tag{19}$$

corresponding to $p = 1$, is reached when $\partial M(x,t)/\partial t = 0$. Thus eq. (19) follows directly from solving $D\partial^2 M_s(x)/\partial x^2 - \alpha M_s(x) + s_0\delta(x) = 0$. Comparing with the full solution in eq. (4) one can see that $M(x,t)$ approaches $M_s(x)$ when $\exp(\pm\beta x)\,\mathrm{erfc}((2\beta Dt \pm x)/\sqrt{4Dt})$ goes to zero. A sufficient condition for this is that $2\beta Dt \pm x \gg \sqrt{4Dt}$, which is fulfilled if

$$t \gg \frac{x}{\beta D} \quad \text{and} \quad t \gg \tau\,. \tag{20}$$

3 Robustness of threshold position

In the main text we highlight the shift in position induced by altering the morphogen profile as described by eq. (12). Here we present the general expression used to derive this result.

A change in the morphogen production rate s_0 by a factor γ results in a modified morphogen profile $M'(x) = \gamma M(x)$. Assume that a certain morphogen concentration M_c defines a position x_c such that $M(x_c) = M_c$. In general, for a different morphogen profile M' this position is shifted to x'_c such that $M'(x'_c) = M_c$. (For a monotonically decreasing profile a solution for x' exists if $M'(0) \geq M(x_t)$ which is assumed in the following.) Thus, the shift is

$$\Delta x \equiv x'_c - x_c = x(M')|_{M'=M_c} - x(M)|_{M=M_c}\,, \tag{21}$$

where $x(M)$ is the inverse function of $M(x)$. Expanding in $\log\gamma = \log M'(x) - \log M(x)$,

$$x(M')|_{M'=M_c} = x(M)|_{M=M_c} + \left.\frac{\partial x}{\partial \log M}\right|_{x=x_c} \cdot \log\gamma + \mathcal{O}(\log^2\gamma) \tag{22}$$

gives

$$\Delta x \simeq \left.\frac{\partial x}{\partial \log M}\right|_{x=x_c} \cdot \log\gamma = \left.\frac{M}{\frac{\partial M}{\partial x}}\right|_{x=x_c} \cdot \log\gamma\,. \tag{23}$$

For the exponential steady-state profile $M_s(x)$ we have $x(M_c) = -\log M_c/\beta + const$ and eq. (23) gives

$$|\Delta x_s| = \frac{\log\gamma}{\beta}\,. \tag{24}$$

Note that the above expression is exact, since all the higher derivatives $\partial^n x(M_c)/(\partial \log M_c)^n$ vanish for $n > 1$. Importantly, the value of Δx_s does not depend on x_c.

For a morphogen profile described by the phenomenological approximation in eq. (12) we find

$$|\Delta x_p(x,t)| \simeq \frac{\lambda(t)}{p(t)} \left(\frac{x}{\lambda(t)}\right)^{1-p(t)} \cdot \log\gamma\,. \tag{25}$$

Finally, for the exact solution in eq. (4) we have ($x > 0$)

$$|\Delta x(x,t)| \simeq \frac{1 - \frac{1}{2}\,\mathrm{erfc}\left(\frac{2\beta Dt - x}{\sqrt{4Dt}}\right) - \frac{e^{2\beta x}}{2}\,\mathrm{erfc}\left(\frac{2\beta Dt + x}{\sqrt{4Dt}}\right)}{1 - \frac{1}{2}\,\mathrm{erfc}\left(\frac{2\beta Dt - x}{\sqrt{4Dt}}\right) + \frac{e^{2\beta x}}{2}\,\mathrm{erfc}\left(\frac{2\beta Dt + x}{\sqrt{4Dt}}\right)} \cdot \frac{\log\gamma}{\beta}\,. \tag{26}$$

We note that the above estimates are not very accurate for $\gamma = 2$ ($\log 2 \simeq 0.7$) and that exact predictions for $\Delta x(x,t)$ were obtained numerically.

4 Numerical solutions

4.1 The time-dependent morphogen gradient in two dimensions

Clearly the one dimensional model discussed above simplifies any real biological morphogen system. In particular, the more complex geometry of the *Drosphila* embryo might affect our quantitative predictions. In order to address this issue we solved eq. (1) in two dimensions using the Matlab© partial-differential-equation (PDE) tool.

We defined a two dimensional field of elliptical shape. The major axis was defined to be of unit length, and the minor axis had length 0.4. The source was defined as the intersection of this ellipse (corresponding to the embryo) and a second small ellipse centered close to the anterior pole of the field (see Suppl. Fig. 2). The boundary conditions were set to be of the Neumann kind, imposing zero flux over the circumference of the large ellipse and unit flux from the source. A fine mesh of ~ 4000 points within the field was generated and the PDE was solved numerically for $D = 0.04$, $\alpha = 1$ and times $t/\tau = 0.1, 0.2, 0.5, 1.0, 2.0$. Subsequently, the solutions on 100 mesh points close to the major axis was extracted. For each solution a polynomial (of degree 15) was fitted to the logged morphogen concentration at these points. The same procedure was applied for halved and doubled morphogen flux. The resulting polynomial approximations to the morphogen profiles for the different fluxes were used to compute the shift Δx according to eq. (21) as a function of position x at various times t (see Suppl. Fig. 3). To control for geometric effects also computed Δx in the same manner, except that the solution was extracted from 100 points along the boundary of the ellipse.

The following comments are in order: (1) The shifts Δx obtained from the numerical solution exhibit the same qualitative behavior as Δx computed from the analytical solution in eq. (4). In particular, at early times Δx decreases as a function of x. (2) At late times the profiles deviate from an exponential at large x due to the finite size of the field. As a consequence the shift Δx actually increases in this regime. (3) We did not observe any significant difference between the solutions extracted along the major axis and the circumference of the ellipse. Thus we conclude that geometric and finite size effects do not alter our main prediction, that the shifts resulting from a change in production rate decrease away from the source when considering the pre-steady state morphogen profile.

4.2 Previous Simulations of dynamic evolution of gap gene expression

In the early development of the *Drosphila* embryo the product of the maternal gene *bicoid* (*bcd*) regulates the downstream "gap genes". In the naive morphogen model the domain of expression for each gap gene is determined by the region where the Bcd concentration is above a certain threshold and where no other gap gene acting as an suppressor is present. Since most gap genes mutually suppress each other this model predicts a set of gap gene expression domains many of which do not overlap with each other.

Clearly such a simple model cannot give an accurate description of the expression domains that develop dynamically. Recently, an in-depth analysis of the dynamics of the gap genes (assuming a steady-state Bcd profile) was presented by Jaeger *et al.* [1, 2]. We reproduced the dynamic development of the gap genes *cad*, *hb*, *Kr*, *gt*, *kni* and *tll* using the same set of differential equations as in [3] and the parameters of "Gene-circuit 28008" (but ignored nucleic divisions and shutdown during mitosis, c.f. [3]). However, simulations for altered *bcd* dosages (corresponding to rescaled Bcd gradients) did not reproduce the domain shifts reported in our main paper. (Indeed, many gap domains failed to develop at all in these simulations, see Suppl. Figs. 4-6.) Thus, we conclude that at the present level of detail the gene circuit model does not explain the robustness of the gap gene system with respect to changes in *bcd* dosage.

In an earlier work Reinitz *et. al.* [4] attributed the anomalous shifts to the contribution of maternal Hunchback (mat-Hb). We re-analyzed this model after extracting the initial mat-Hb distribution graphically from Fig. 1 of this paper. For simplicity we did not model the nuclear divisions, but assumed 64 nuclei from the start. Our re-analysis generated the correct gap gene expression domains (Suppl. Fig. 7). Changes in *bcd* dosage (by amplitude scaling) revealed that the model predicts correctly reduced sensitivity of the Hb position to one or three bcd copies. Yet, it suffered from several drawbacks: First, inconsistent gap gene patterns arose when mat-Hb was set to zero (Suppl. Fig. 8), in contrast to experimental results, which show that deletion of mat-Hb has no effect on patterning. Second, also for four bcd copies this model did not produce a consistent set of gap gene domains (Suppl. Fig. 9). Third, this model did not predict correctly the shift of other gap gene expression domains (Suppl. Fig. 10).

4.3 Our simulations for gap gene expression in response to a time-dependent Bcd gradient

In order to simulate the dynamic response of the gap genes to a Bcd gradient that has not yet reached its steady state we used a simplified toy model that allows for the gradual production, diffusion and mutual repression of the gap genes. For simplicity, we did not include details like maternal gene distributions and nuclei divisions. Rather, our model is based on a system of partial differential equations governing the evolution in time and space of the concentrations of both the morphogen $C_0(x,t) = M(x,t)$ (as described by eq. 1) and N_G downstream gap-genes $C_i(x,t)(i = 1, ..., N_G)$:

$$\frac{d}{dt}C_i(x,t) = D_i \frac{\partial^2}{\partial x^2} C_i(x,t) - \alpha_i C_i(x,t) + s_i(t) \,. \tag{27}$$

Here D_i and α_i refer to the diffusion constant and degradation rate of gene i. The interactions between the gap genes and their dependence of on the Bcd concentration are encoded in the source term:

$$s_i(t) = \Theta(t - t_i) p_i \cdot \sum_{j \in A_i} h(C_i/\tilde{C}_i^{(j)}, n_i^{(j)}) \cdot \prod_{j \in S_i} h(C_i/\tilde{C}_i^{(j)}, n_i^{(j)}) \,, \tag{28}$$

where t_i is the initiation time and p_i refers to the production rate of gene i. We use the sum of Hill functions

$$h(y,n) \equiv \frac{y^n}{1 + y^n} \tag{29}$$

for the activating genes A_i, which is multiplied by the Hill functions of the suppressing genes S_i. Specifically, for each gap gene i there is a critical value $\tilde{C}_i^{(j)}$ for the concentration of gene j, where the hill function equals to 1/2, the transition point between suppression ($h = 0$) and activation ($h = 1$). The magnitudes of the powers $n_i^{(j)}$ dictate how rapid this transition is, and the signs determine if below the transition point the gene is suppressed ($n < 0$) or activated ($n > 0$). We note that the additive integration used for the activators corresponds to an OR-gate while the multiplication of the suppressing terms is similar to an AND-gate.

The principle question we address here is whether there exists a set of parameters $\{t_i, p_i, \alpha_i, \tilde{C}_i^{(j)}, n_i^{(j)}\}$ for which the expression domains of the gap genes are determined by the pre-steady state Bcd profile and remain fixed when the Bcd gradient approaches its steady state shape. Intuitively such a scenario could be achieved due to the mutual suppression of the gap genes: If the expression of the gap genes is initiated before Bcd has reached its steady state the boundaries of the gap gene expression domains in principle could be fixed by neighboring gap genes even though the Bcd concentration continues to change.

Studying the numerical solutions for a wide range of parameters we find that the above scenario can indeed be achieved if

1. The morphogen acts as activator of the gap genes and more posterior target genes have a lower activation threshold, i.e. $n_i^{(0)} > 0$ and $\tilde{C}_1^{(0)} > \tilde{C}_2^{(0)} > ... > \tilde{C}_{N_G}^{(0)}$.

2. Pairs of mutually exclusive gap genes (i, j) suppress each other with the more anterior gene being dominant, i.e. $n_i^{(j)}, n_j^{(i)} < 0$ and $\tilde{C}_i^{(j)} < \tilde{C}_j^{(i)}$.

3. The gap gene production rates are significantly larger than the Bcd production rate, i.e. $p_i \gg p_0 = s_0$.

4. The gap gene decay rates are significantly larger than the Bcd decay rate, i.e. $\alpha_i \gg \alpha_0 = \alpha$.

5. The gap gene diffusion constants are much smaller than the Bcd diffusion constant, i.e. $D_i \ll D_0 = D$.

Condition (1) and (2) are required to induce the gap gene expression in different domains. Conditions (3) and (4) ensure a rapid response of the gap gene system, such that the gap genes integrate positional information from the transient Bcd gradient over a relatively short period of time. Condition (5) is needed to immobilize the genes products once they have accumulated in certain position.

Suppl. Fig. 11 shows the time evolution of the morphogen gradient for the following choice of parameters: $t_0 = 0, t_i = 1, p_0 = 1, p_i = 20, \alpha_0 = 1, \alpha_i = 20, D_0 = 1, D_i = 10^{-2}, n_i^{(0)} = 4, n_i^{(j)} = -4, \tilde{C}_i^{(0)} = M(x_i, t_i), \tilde{C}_i^{(j)} = 10, 20, 30$ for $i <, =, > j$, respectively. The positions of the gap gene expression boundaries depended most strongly on the parameters x_i which were chosen to fit the experimental results for wt embryos (but kept fixed for altered *bcd* dosage). It is important to note that such a toy model can only demonstrate in principle that it is possible to fix the expression domains of the gap genes according to the pre-steady state Bcd profile and shed some light on the required qualitative relations between its parameters. We did not attempt to fit all parameters to fully reproduce all details of the gap gene expression domains.

We note that for our theoretical analysis we prefer to work in natural units given in terms of the steady state decay length λ and the decay time τ. In the context of the Bcd morphogen system these units are set by the properties of the system. Specifically, $\lambda \simeq 75\mu m$ in *Drosophila melanogaster* embryos. Moreover, the prediction of our model for Δx fit the experimental data for $t_i \simeq \tau$. Since gap gene expression is initiated at $t_i \sim 90$ minutes, this fixes the decay time to this scale. Note that this implies that the half-life time is $t_{1/2} = log(2) \cdot \tau \sim 1h$ and the Bcd diffusion constant is $D \sim 1\mu m^2/sec.$)

For comparison we reproduced Fig. 1, but setting $D = 10\mu m^2/sec$ such that patterning occurs essentially after the Bcd morphogen gradient has reached its steady state (Suppl. Fig. 12). One can see that the reduced sensitivity to changes in *bcd* dosage (as observed for pre-steady state decoding) is lost.

5 The effect of morphogen binding

The morphogen activates its target genes by binding to their regulatory elements. Since the morphogen cannot diffuse in the bound state a more accurate description of the morphogen profile should include not only the free morphogen $M(x,t)$, but also its bound form $M_b(x,t)$. The corresponding coupled reaction-diffusion equations read:

$$\frac{d}{dt}M = D\frac{\partial^2}{\partial x^2}M - \alpha M + k_{-b}M_b - k_b MB\,, \tag{30}$$

$$\frac{d}{dt}M_b = -k_{-b}M_b + k_b MB\,, \tag{31}$$

where $B(x,t) = B_{tot}(x,t) - M_b(x,t)$ is the density of *free* binding sites (the difference between the total and the occupied sites) and k_b (k_{-b}) is the rate of (un)binding. Here we assume that these binding sites do not diffuse and we suppress the source terms.

First note that morphogen binding to receptors can only affect the morphogen gradient, as long as the system has not yet reached its steady state, since for $dM_b/dt = 0$ eqs. (30) and (31) reduce to eq. (1).

Let us assume that the binding and unbinding reaction proceeds much faster than the diffusion and decay of the morphogen (corresponding to $k_{-b} \gg \alpha$ and $k_b B \gg \alpha$) Given this separation of time scales for a *given* $M(x,t)$ the change in time of $M_b(x,t)$ is negligible. Assuming that we have a such a quasi-steady-state

$$\left.\frac{dM_b}{dt}\right|_M = k_{-b}M_b|_M - k_b MB \simeq 0\,. \tag{32}$$

one finds that

$$M_b|_M = B_{tot}\cdot\frac{M}{K+M}\,, \tag{33}$$

where $K \equiv k_{-b}/k_b$.

The total change in time of M_b is then predominantly due to the change in M, i.e.:

$$\frac{dM_b}{dt} = \left.\frac{dM_b}{dM}\right|_M \cdot \frac{dM}{dt} = \frac{B_{tot}\cdot K}{(K+M)^2}\cdot\frac{dM}{dt}\,. \tag{34}$$

Thus, summing up eqs. (30) and (31) one finds

$$\frac{dM}{dt} + \frac{dM_b}{dt} = \left(1 + \frac{B_{tot}\cdot K}{(K+M)^2}\right)\cdot\frac{dM}{dt} = D\frac{\partial^2}{\partial x^2}M - \alpha M\,. \tag{35}$$

Consequently, morphogen binding effectively reduces the time-scale of the reaction-diffusion equation (1). In particular, for $M \ll K$ one has $M_b|_M \simeq M\cdot B_{tot}/K$. In this linear regime

$$\frac{dM_b}{dt} = \frac{B_{tot}}{K}\cdot\frac{dM}{dt}\,, \tag{36}$$

corresponding to a trivial re-scaling

$$t \to \tilde{t} = \frac{t}{1 + B_{tot}/K} \,. \tag{37}$$

In the early *Drosophila* development the number of nuclei is doubled in each cleavage cycle, $B_{tot} \sim 2^{t/t_{cycle}}$. The "slowing down" effect induced by the morphogen binding becomes significant as soon as $B_{tot} \gtrsim K$. Importantly, the above analysis shows that morphogen binding does not alter our main result that the positional decoding of pre-steady gradient decreases sensitivity to production rate. Rather, this effect has the potential to increase the time it will take to reach the steady state gradient.

6 Details on transition point and nuclei density measurements

The two corresponding sets of images were analyzed with automated image processing tools developed in MatLab in order to measure the the nuclei density as proxy for the embryo's age (Suppl. Fig. 13) and the transition point of *lac*Z expression (Suppl. Fig. 14).

To establish the transition point of the *lac*Z gradient, a software interface was designed to mark the anterior and posterior poles (defining the A-P axis) and two additional points (setting the region in which the transition point lies). Based on this input points and predefined margins a band was extraced from the image for further analysis. Specifically, the following computations were performed on this band: (a) for each point along the A-P axis a mean pixel intensity was defined by averaging over all point in the band perpendicular to the AP axis. (b) The resulting profile was normalized to the range [0,1]. (c) Using this rescaled profile, the transition point was defined as the left boundary of a given interval possessing the mean value of 0.5 (half-point).

To calculate nuclear density, a rectangle was defined on digital images of embryos stained for their nuclei. Nuclei were then automatically counted (using standard segmentation algorithms), and the total number was divided by the rectangle area, to obtain the nuclear density value. We used the log2-values of the ratio between the nuclei density of the respective image and that from embryos on the same slide that had already reached cycle 14. Finally, to assess the extent of Bcd gradient movement, nuclear densities were correlated with the corresponding transition point of the Bcd-responsive *lac*Z gradient. In addition, transition point values were clustered according to geometric distances between their corresponding nuclear density values, so that each cluster represented a nuclear division cycle. For each cluster the mean and std were calculated and compared to each other using a Kolmogorov-Smirnov. The nominal p-value for obtaining the measured transition points for cycle 11 and 12 from the same probability distribution is $p < 10^{-8}$.

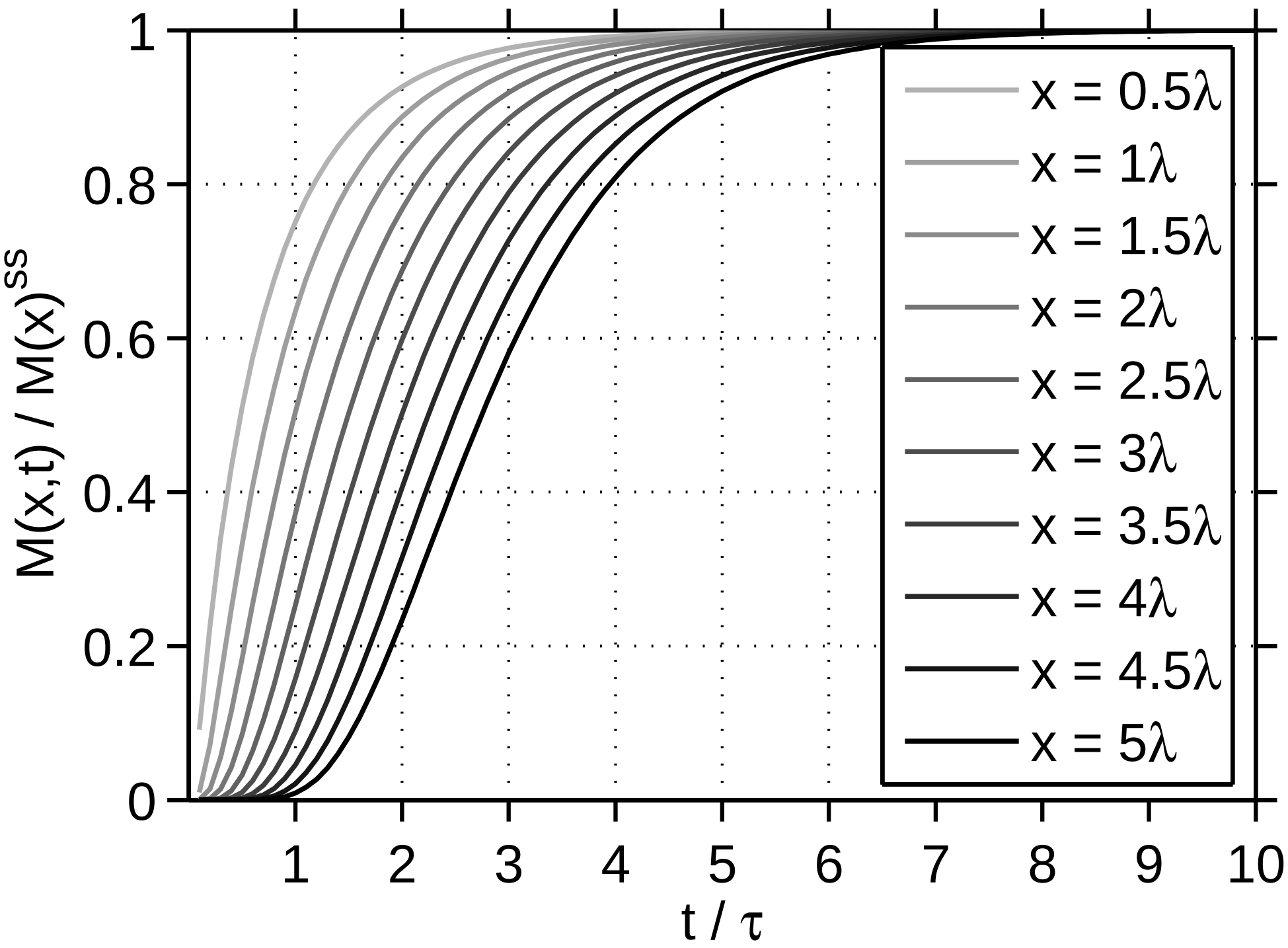

Supplementary Figure 1: The ratio between the morphogen concentration $M(x,t)$ and its steady state value $M(x)^{ss}$ is plotted as a function of time t (in units of the decay time τ) at different positions x (in units of the decay length λ). Note that close to the source (at $x = 0$) the morphogen gradient approaches its steady state value much earlier than far away from the source.

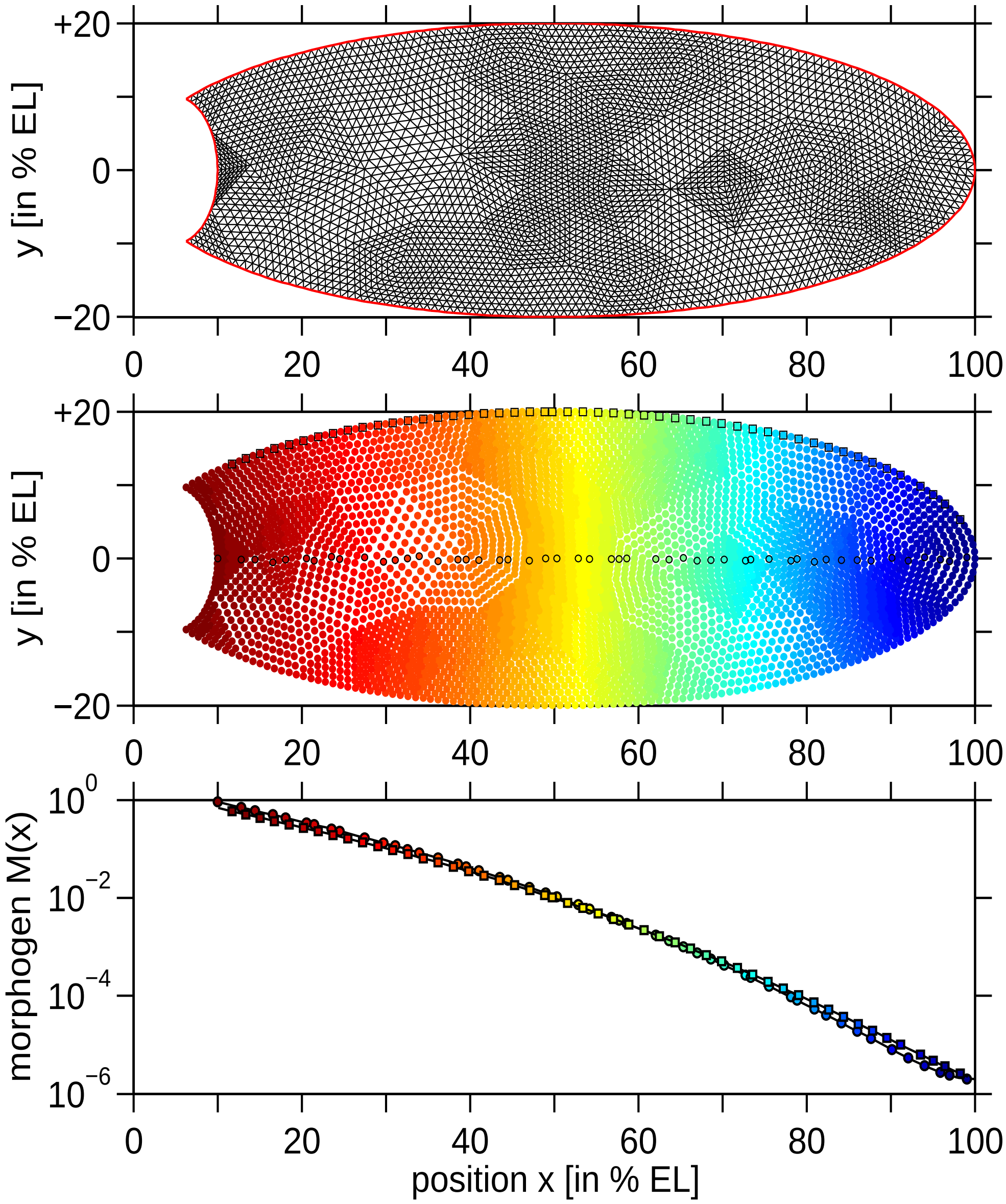

Supplementary Figure 2: (a) A mesh with $\sim$4000 points defining a two-dimensional field of elliptical shape was generated using Matlab. The major axis of the ellipse was set to unitlength, and the minor axis to 40% unitlength. (b) Eq. (1) for $D = 0.04$ and $\alpha = 1$ was solved numerically on this mesh at different times t and for different morphogen fluxes. Shown here is the two-dimensional solution as a function of the coordinates x and y at $t = \tau$. The solutions on the mesh points close to the major axis (circles) and along the boundary (squares) were extracted. (c) The corresponding profiles $M(x)$ have very similar shape, in particular in the center of the elliptical field.

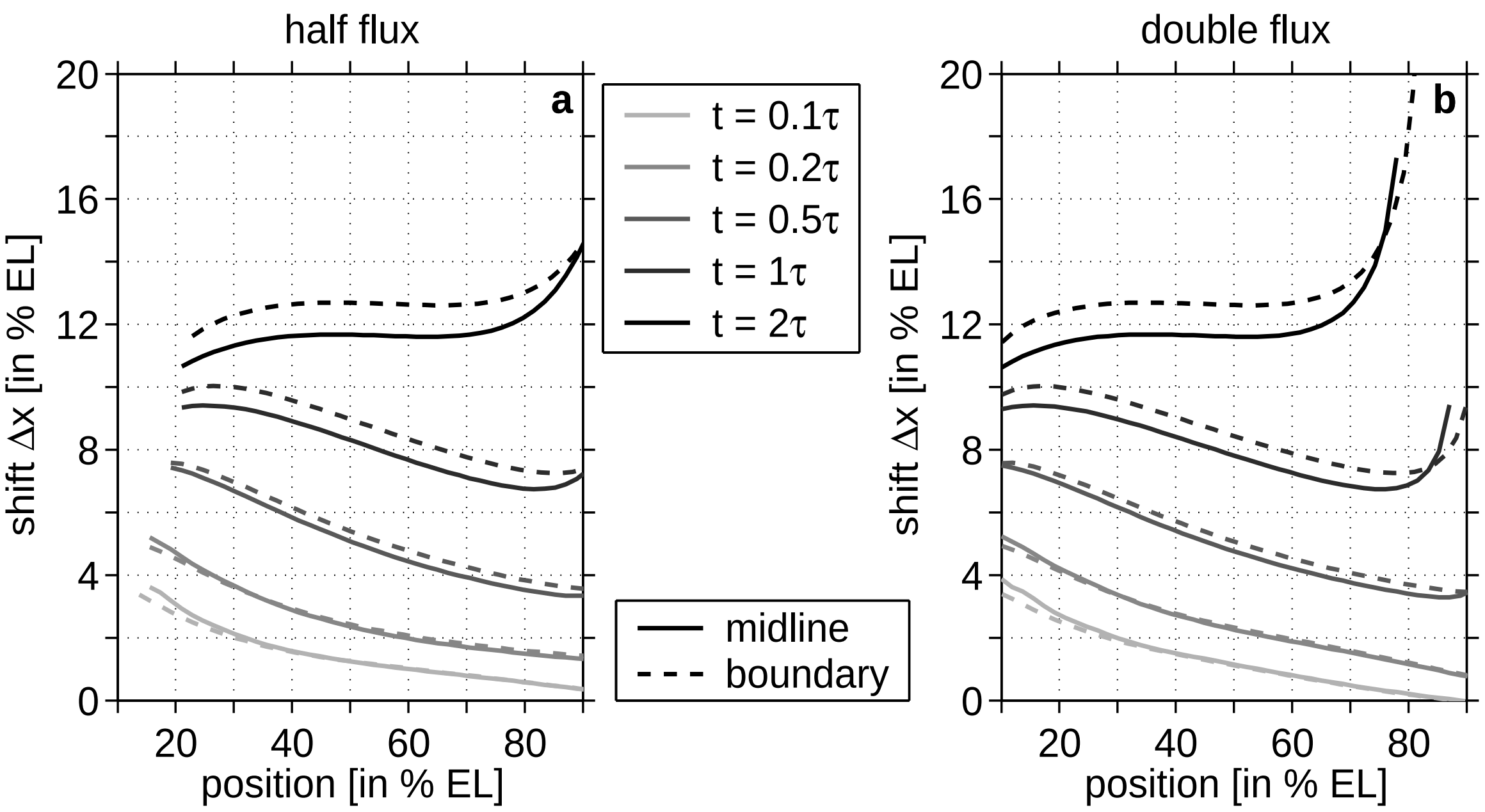

Supplementary Figure 3: Profiles for half, unit and double flux where extracted from the two-dimensional solutions at different times (legend) as described in Suppl. Fig. 1. Polynomial approximations to the morphogen profiles from the mesh points along the midline (solid) and the boundary (dashed) of the elliptical field were used to compute the shift Δx according to eq. (21) as a function of position x. The shifts resulting from halving (a) and doubling (b) the morphogen flux exhibit the same qualitative behavior as Δx computed from the analytical solution in eq. (4), i.e. at early times Δx decreases as a function of x. Geometric and finite size effects do not alter this behavior significantly, except for the region opposite to the source ($x \gtrsim 75\%$ EL).

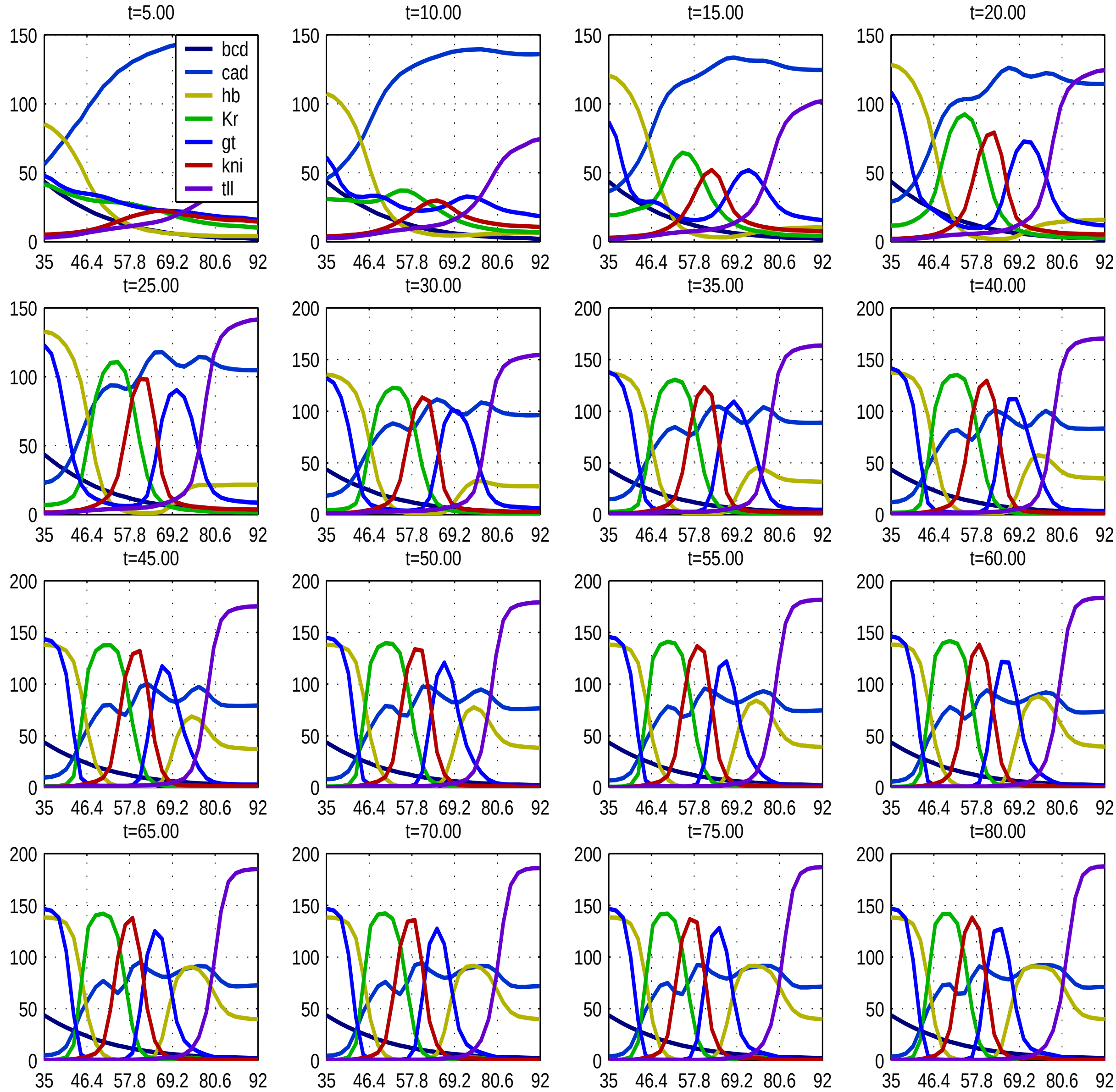

Supplementary Figure 4: Numerical simulation of the gap genes system using the gene circuit model by Reinitz and colleagues. The Bcd gradient is stationary. Note that we reproduced the dynamic development of the gap genes *cad*, *hb*, *Kr*, *gt*, *kni* and *tll* using the same set of differential equations and parameters of "Gene-circuit 28008" but ignoring nucleic divisions and shutdown during mitosis. The x-axes denote %EL and the y-axes refer to the concentrations of the various gene products.

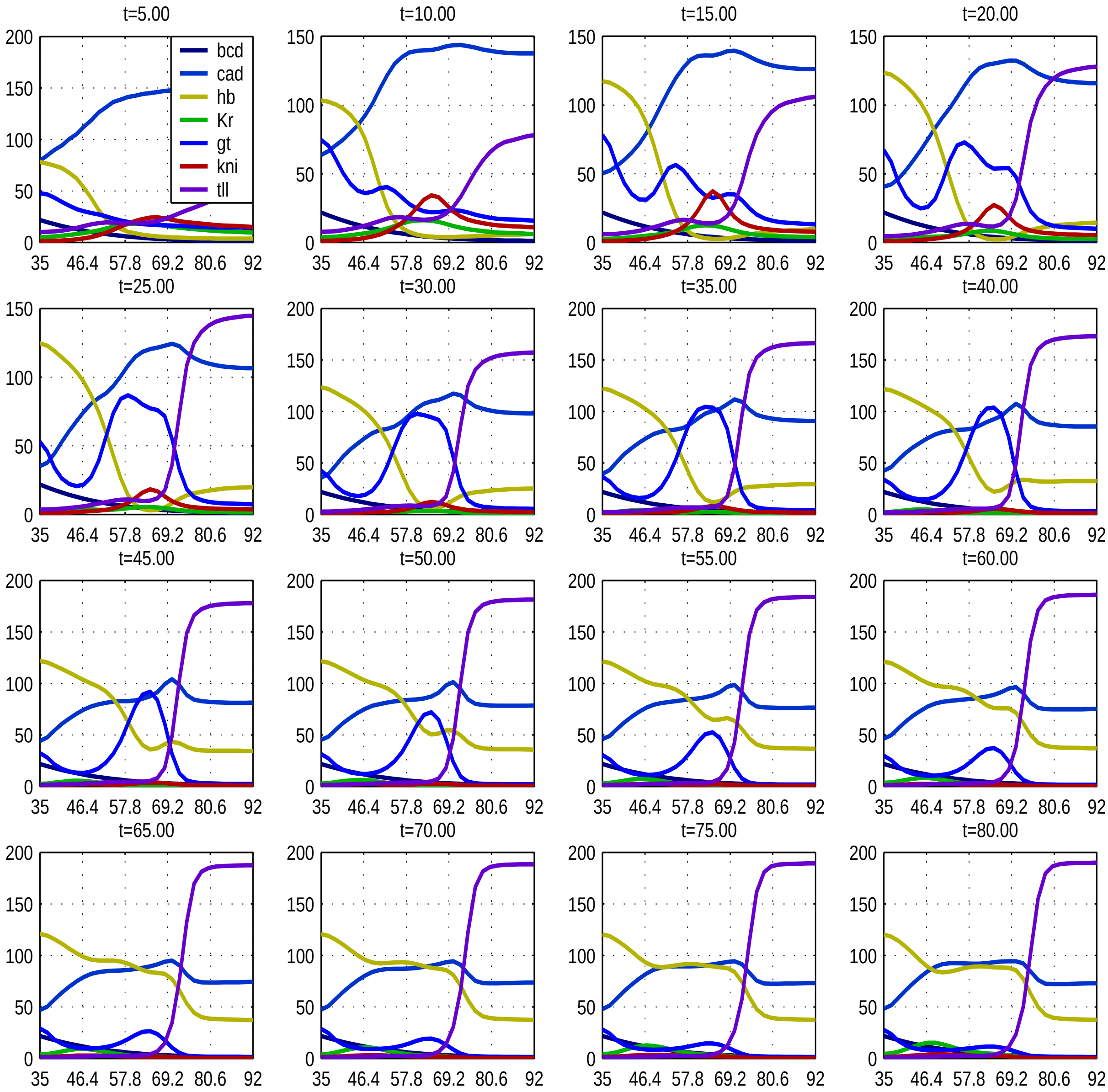

Supplementary Figure 5: Numerical simulation of the gap genes system using the gene circuit model by Reinitz and colleagues but for half *bcd* dosage (corresponding to a Bcd profile that is half of the wildtype). Note that only the Cad, Hb and Tll domains develop.

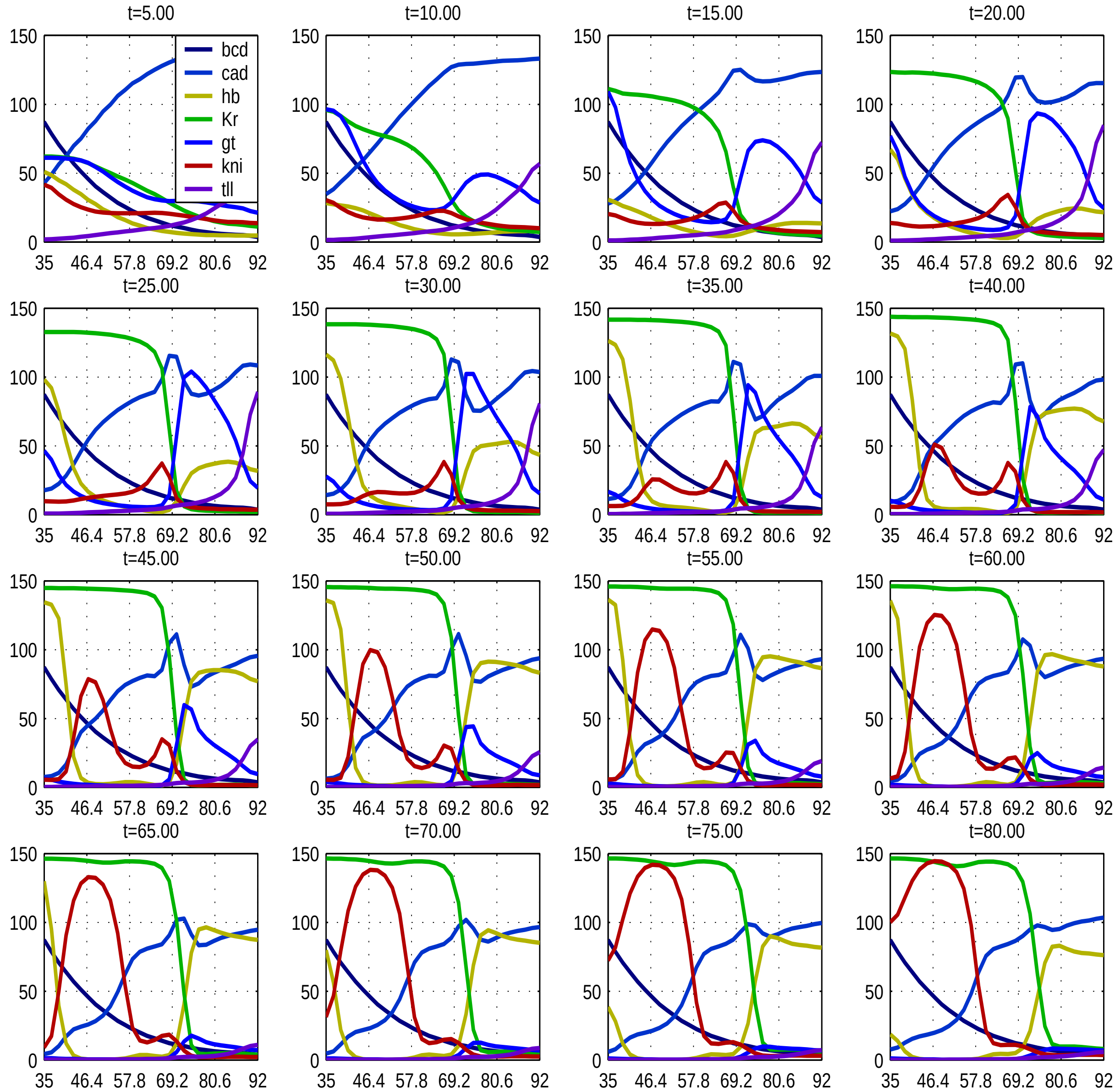

Supplementary Figure 6: Numerical simulation of the gap genes system using the gene circuit model by Reinitz and colleagues but for double *bcd* dosage (corresponding to a Bcd profile that is double of the wildtype). Note that only the Kni, Kr, Cad, and Hb domains develop.

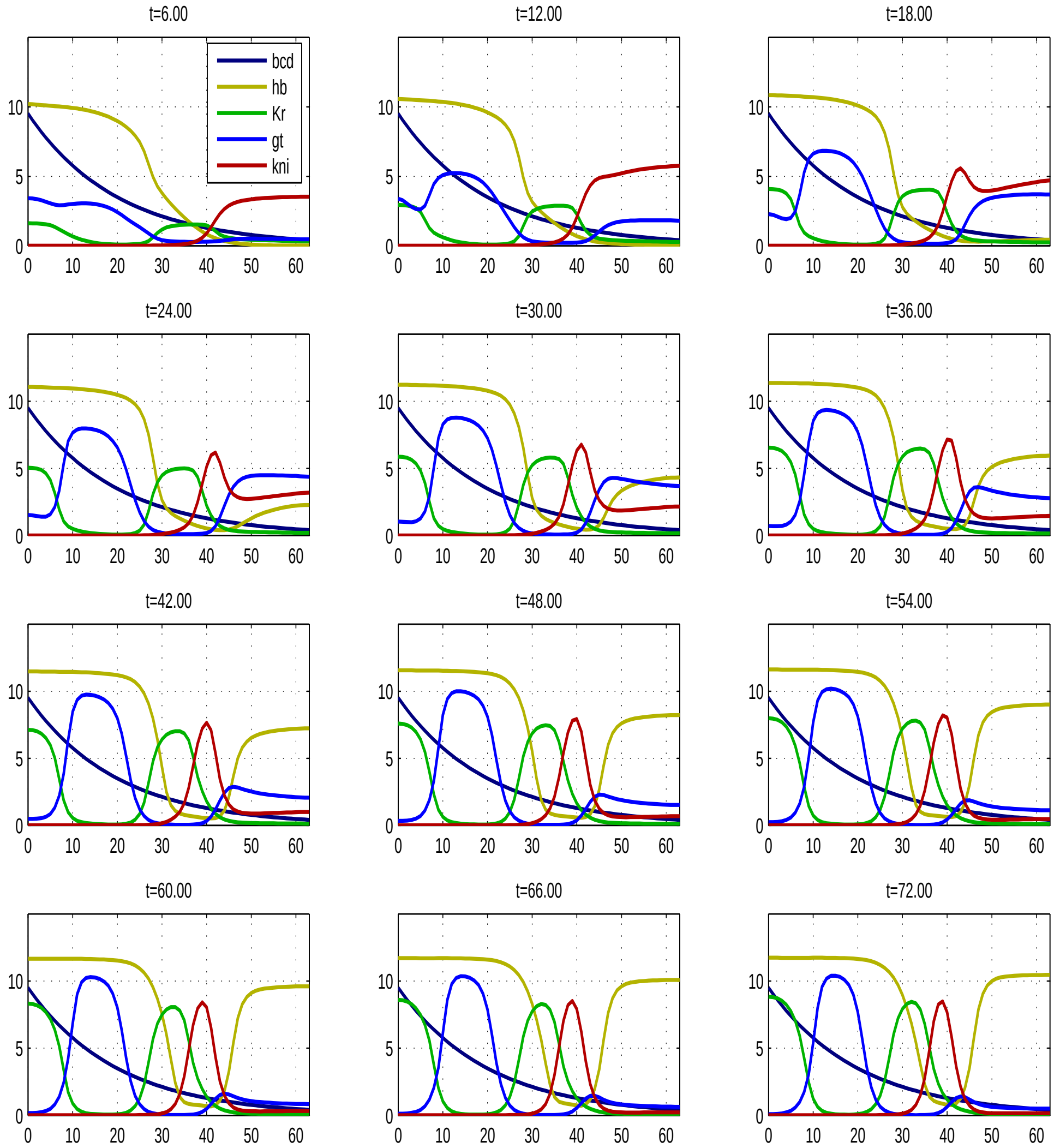

Supplementary Figure 7: Numerical simulation of the gap genes system using the gene circuit model proposed by Reinitz and colleagues in 1995 (for a Bcd profile that corresponds to the wildtype). Note that our simplified re-analysis generates the correct gap gene expression domains.

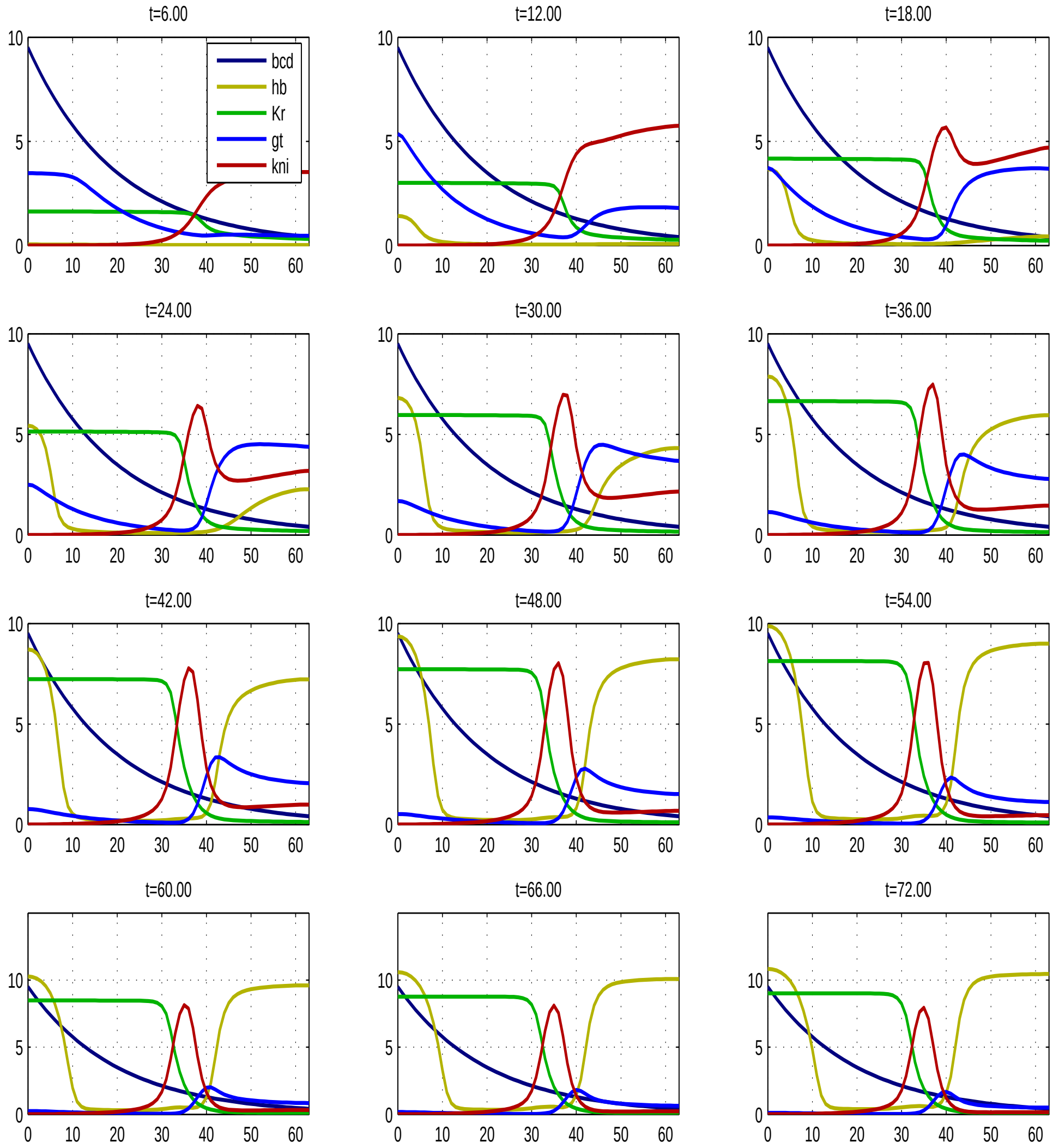

Supplementary Figure 8: Numerical simulation of the gap genes system using the gene circuit model proposed by Reinitz and colleagues in 1995 (using the optimal parameters for the model including mat-Hb, but setting mat-Hb to zero everywhere). Note that the gap gene expression domains do not develop correctly.

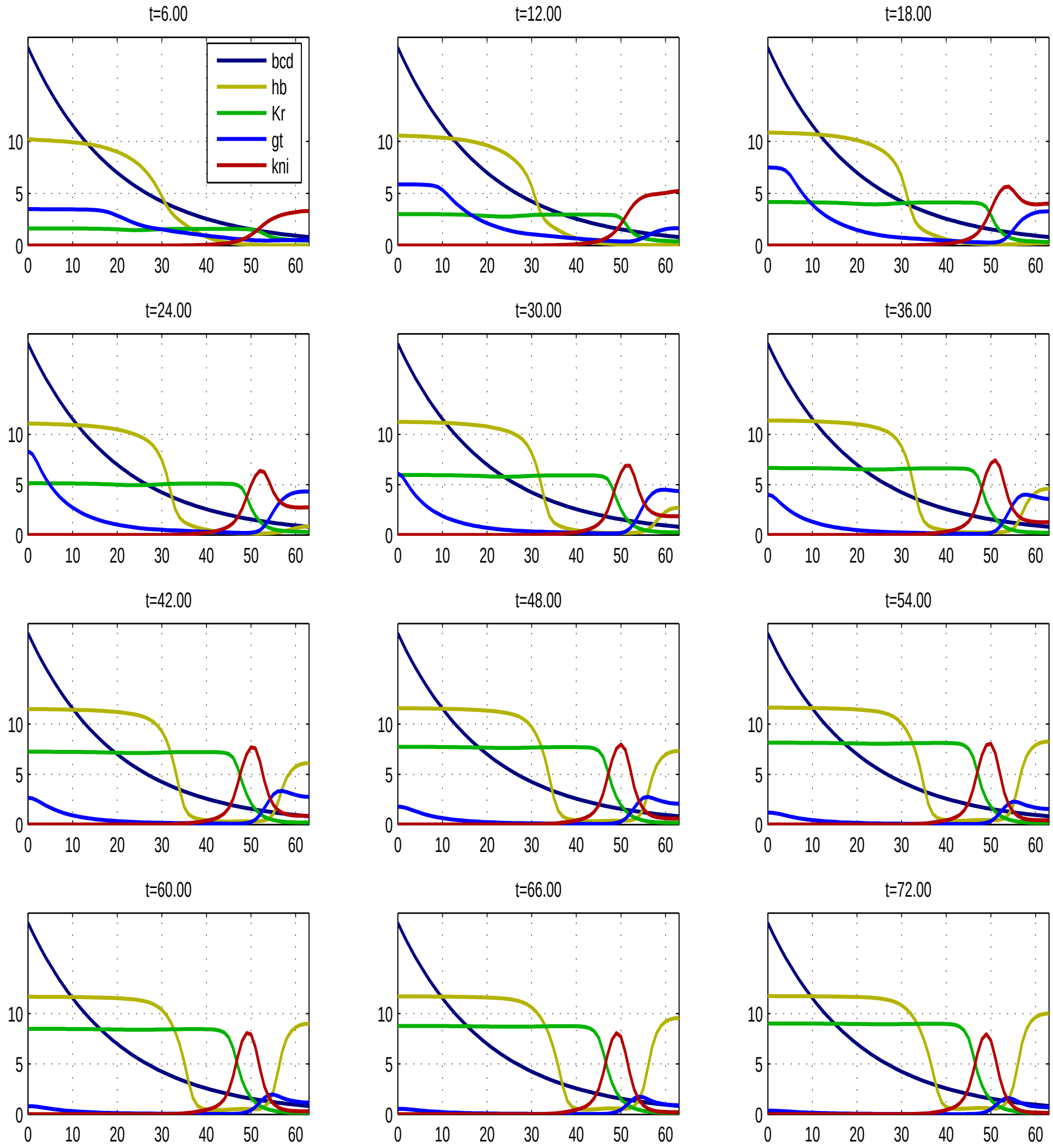

Supplementary Figure 9: Numerical simulation of the gap genes system using the gene circuit model proposed by Reinitz and colleagues in 1995 (for a Bcd profile that corresponds to double gene dosage of the wildtype, i.e. 4x *bcd*). Note that our re-analysis does not generate the correct gap gene expression domains.

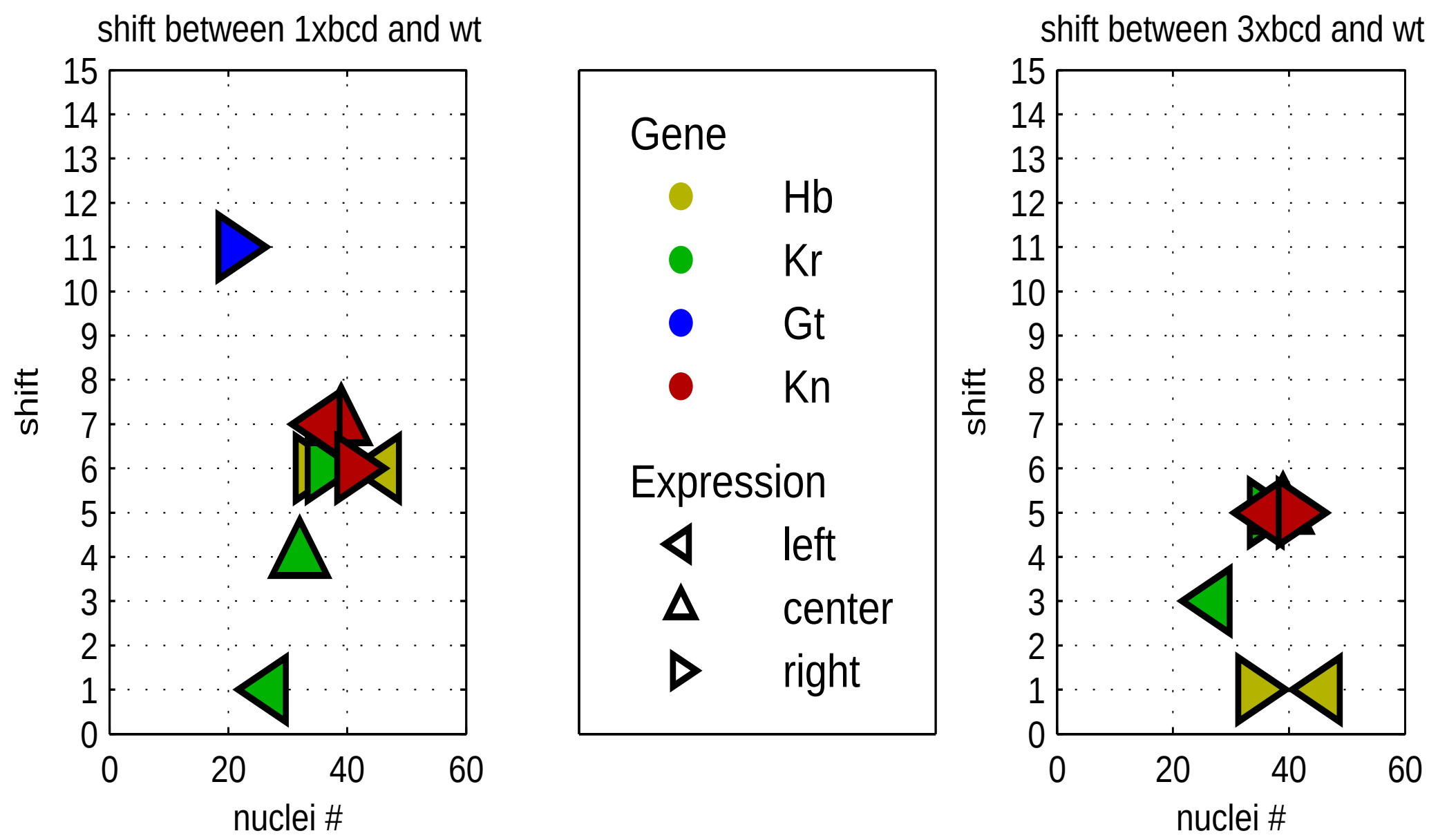

Supplementary Figure 10: Predicted positional shifts of gap gene expression domains for reduced (1x *bcd*) and increased (3x *bcd*) morphogen production rate according to our re-analysis of the gene circuit model proposed by Reinitz and colleagues in 1995 (using the optimal parameters for the model including mat-Hb, and applying amplitude scaling to the Bcd profile). Note while the Hb shifts are indeed relatively small in this model, the positional shifts of other genes are not consistent with our experimental observations. In particular the magnitude of the shifts does not decrease towards the posterior pole.

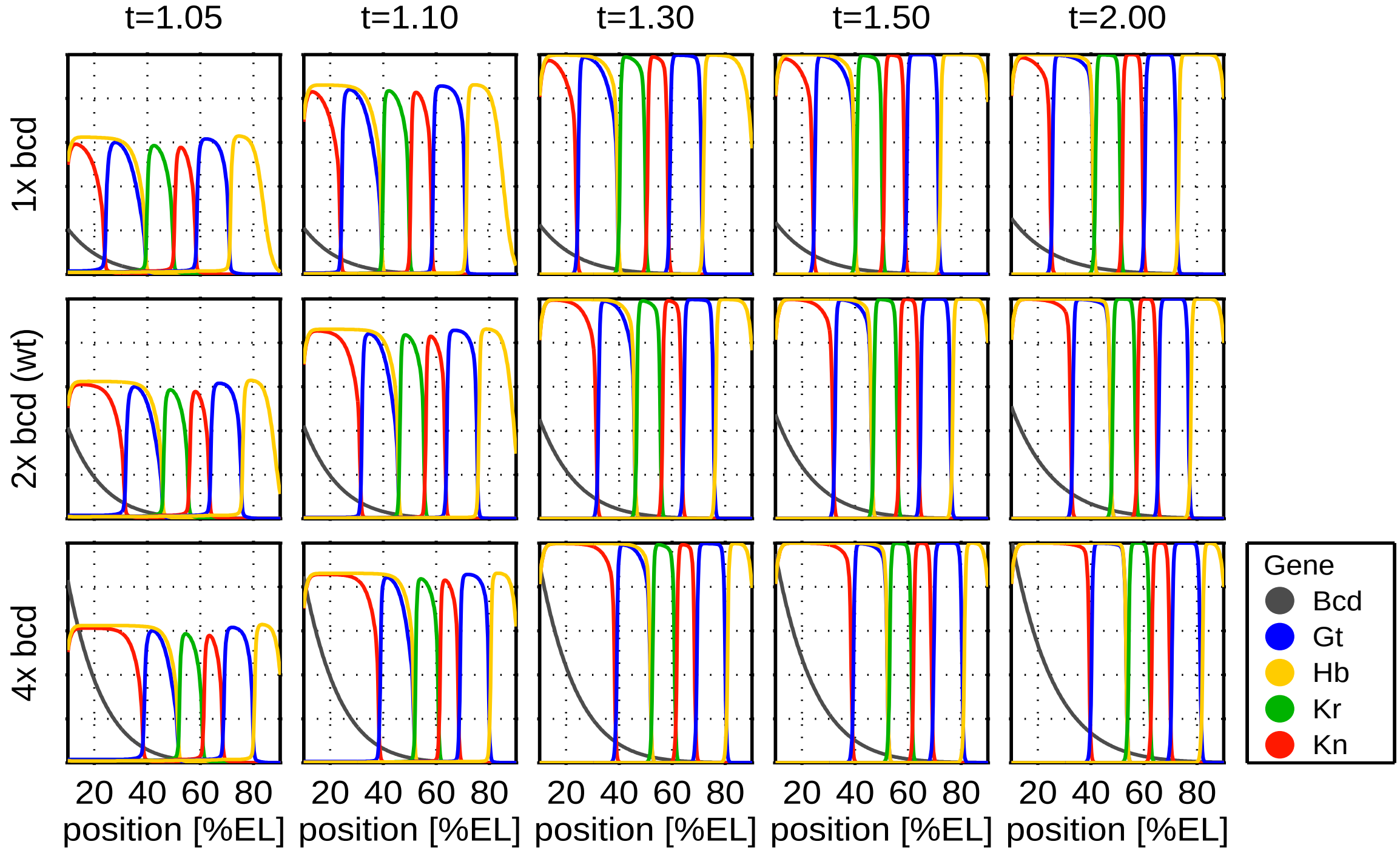

Supplementary Figure 11: Time course of the expression profiles according to our *in-silico* dynamical simulation of the gap gene network. Note that our model allows for positional decoding of the Bcd morphogen profile before it has reached its steady state (see text for details of the model). Gap gene expression is initiated at $t_i = 1$ with a production and degradation rates 20 times larger than that of Bcd, while gap gene diffusion is negligible with respect to Bcd ($D_i = 10^{-2}D$). The gap gene activation function is determined by $n_i^{(M)} = 4$ and a hierarchy of critical densities, $\tilde{M}_i = M(x_i, t_i)$, while the mutual gap gene suppression is due to $n_i^{(j)} = -4$ and $\tilde{G}_i^{(j)} = 10, 20, 30$ for $i <, =, > j$, respectively.

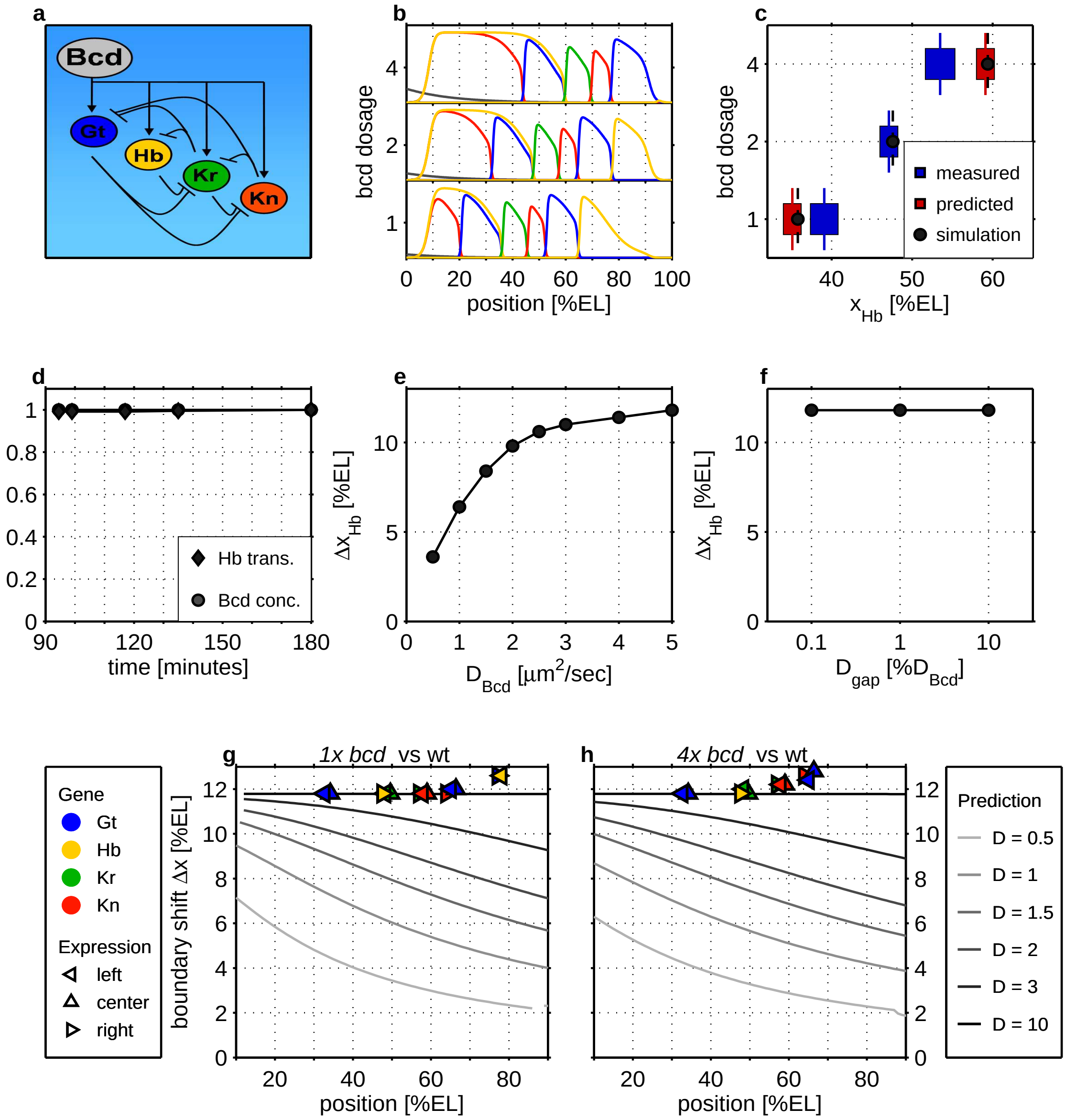

Supplementary Figure 12: Same as Fig. 1 of the paper, but setting $D = 10$. For such high Bcd diffusion patterning occurs essentially after the Bcd profile has reached its steady state. Accordingly reduced sensitivity to changes in *bcd* dosage (as observed for pre-steady state decoding) is lost.

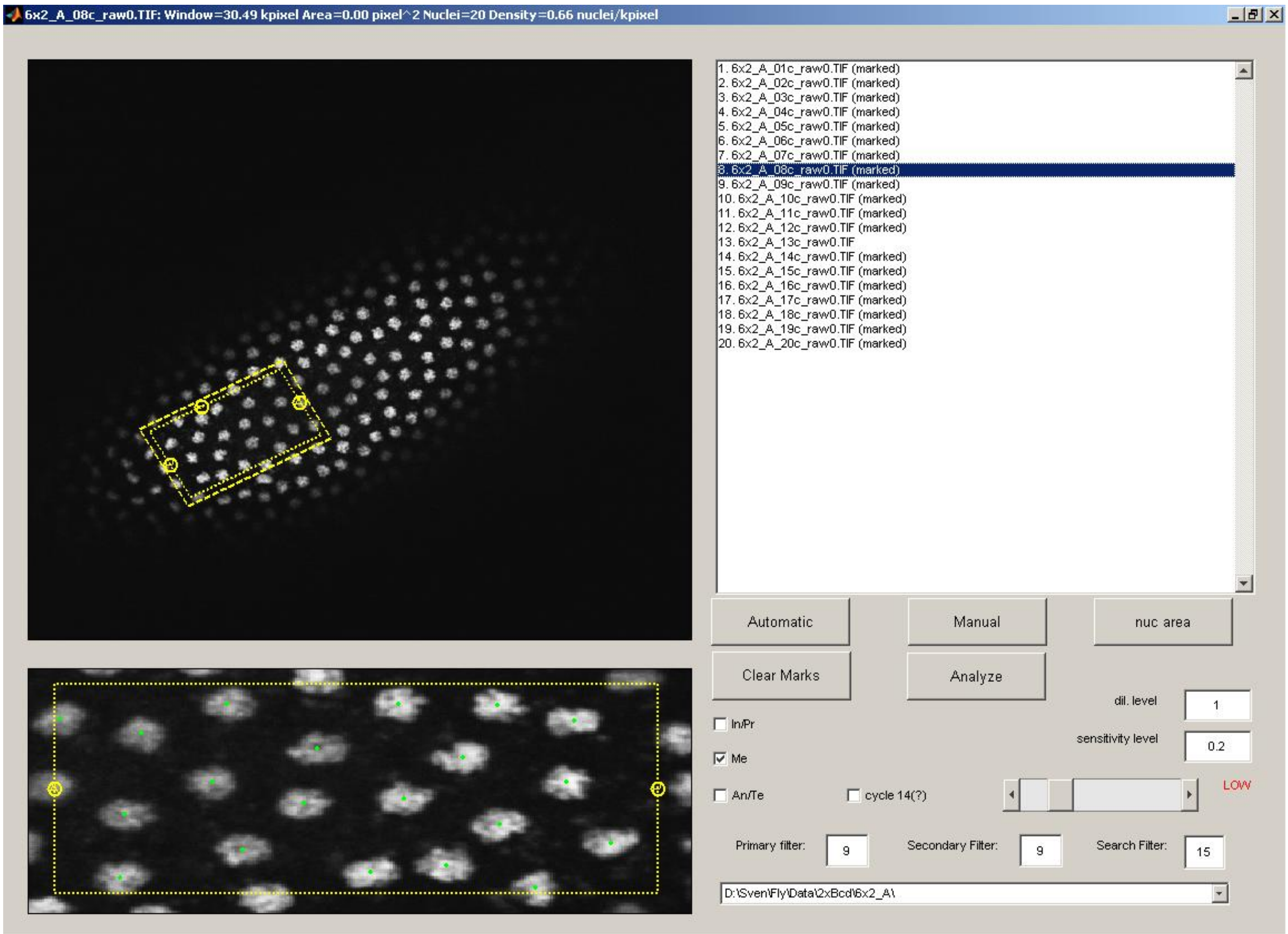

Supplementary Figure 13: Graphic user-interface for analysis of the nuclei density of *Drosophila* embryos.

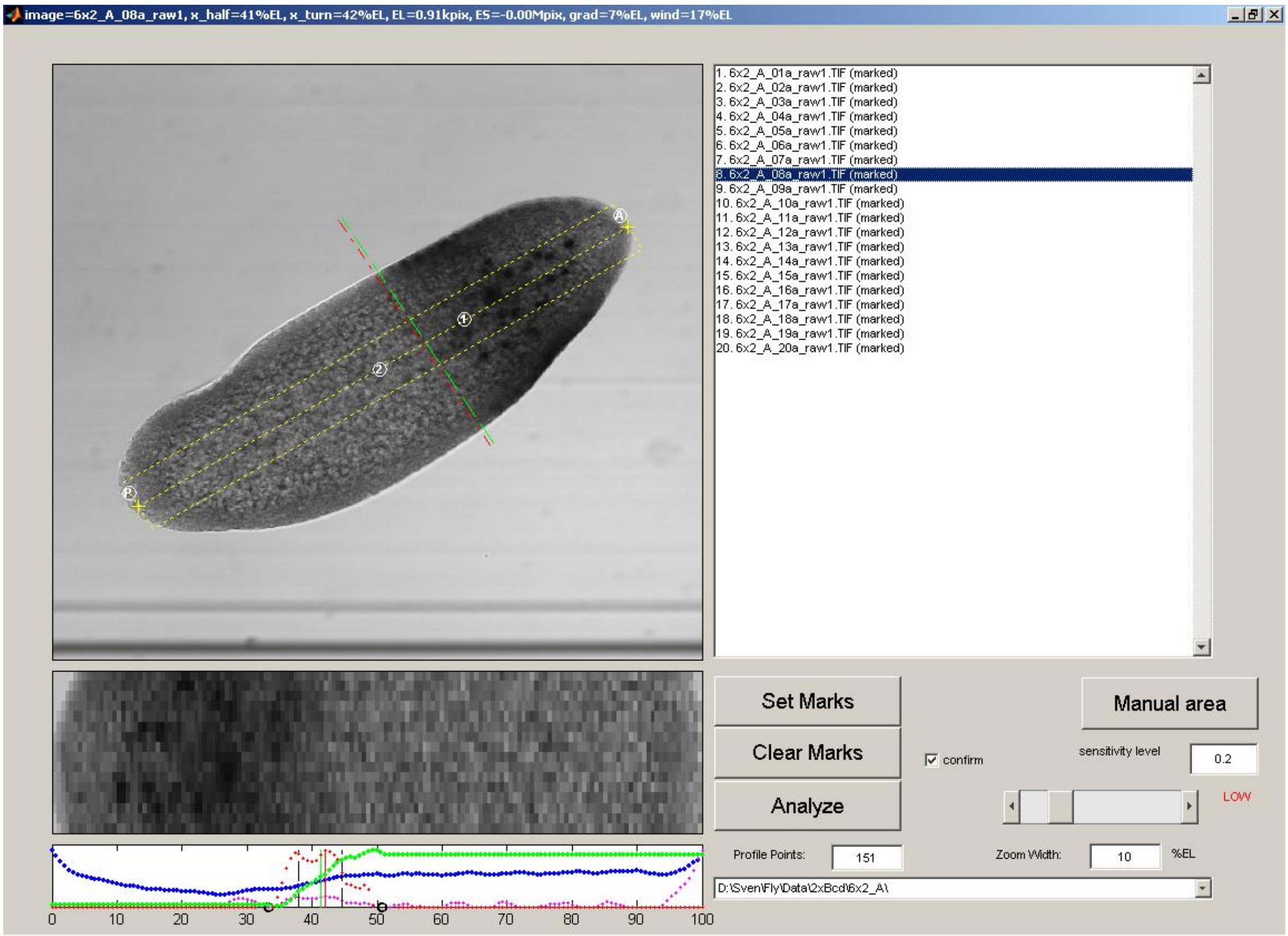

Supplementary Figure 14: Graphic user-interface for analysis of the transition *lacZ* expression boundary.

References

[1] Jaeger J, Blagov M, Kosman D, Kozlov KN, Manu, Myasnikova E, Surkova S, Vanario-Alonso CE, Samsonova M, Sharp DH, Reinitz J., *Dynamical analysis of regulatory interactions in the gap gene system of Drosophila melanogaster*, Genetics 2004 Aug; 167(4):1721-37.

[2] Jaeger J, Surkova S, Blagov M, Janssens H, Kosman D, Kozlov KN, Manu, Myasnikova E, Vanario-Alonso CE, Samsonova M, Sharp DH, Reinitz J., *Dynamic control of positional information in the early Drosophila embryo*, Nature 2004 Jul 15; 430(6997):368-71.

[3] Gurskya VV, Jaeger J, Kozlovc KN, Reinitz J, Samsonova AM, *Pattern formation and nuclear divisions are uncoupled in Drosophila segmentation: comparison of spatially discrete and continuous models*, Physica D 197 (2004): 286-302.

[4] Reinitz J, Mjolsness E, Sharp DH, *Model for cooperative control of positional information in Drosophila by bicoid and maternal hunchback*, J Exp Zool 271(1): 47-56.
